# Mental Health Care Utilization and Prescription Rates Among Children, Adolescents, and Young Adults in France

**DOI:** 10.1001/jamanetworkopen.2024.52789

**Published:** 2025-01-07

**Authors:** Guillaume Fond, Vanessa Pauly, Yann Brousse, Pierre-Michel Llorca, Samuele Cortese, Masoud Rahmati, Christoph U. Correll, Corentin J. Gosling, Michele Fornaro, Marco Solmi, Lee Smith, Nicola Veronese, Dong Keon Yon, Pascal Auquier, Antoine Duclos, Laurent Boyer

**Affiliations:** 1 CEReSS, Research Centre on Health Services and Quality of Life, Aix Marseille University, Marseille, France; 2 FondaMental Fondation, Creteil, France; 3 Université Clermont Auvergne, Centre National de la Recherche Scientifique (CNRS), Clermont Auvergne Institut National Polytechnique (INP), Institut Pascal, Centre Hospitalier Universitaire de (CHU) Clermont-Ferrand, Psychiatry, Clermont-Ferrand, France; 4 Centre for Innovation in Mental Health, School of Psychology, Faculty of Environmental and Life Sciences, University of Southampton, Southampton, United Kingdom; 5 Clinical and Experimental Sciences (CNS and Psychiatry), Faculty of Medicine, University of Southampton, Southampton, United Kingdom; 6 Hampshire and Isle of Wight Healthcare National Health Service Foundation Trust, Southampton, UK; 7 Hassenfeld Children’s Hospital at New York University Langone, New York University Child Study Center, New York City; 8 DiMePRe-J-Department of Precision and Rigenerative Medicine-Jonic Area, University of Bari “Aldo Moro,” Bari, Italy; 9 Developmental EPI (Evidence Synthesis, Prediction, Implementation) Lab, Centre for Innovation in Mental Health, School of Psychology, Faculty of Environmental and Life Sciences, University of Southampton, Southampton, UK; 10 Department of Child and Adolescent Psychiatry, Universitätsmedizin Berlin, Berlin, Germany; 11 Departments of Psychiatry and Molecular Medicine, Zucker School of Medicine at Hofstra/Northwell, Hempstead, New York; 12 Department of Psychiatry, Zucker Hillside Hospital, Northwell Health, Glen Oaks, New York; 13 DysCo Lab, Department of Psychology, Université Paris Nanterre, Nanterre, France; 14 Laboratoire de Psychopathologie et Processus de Santé, Université de Paris, Boulogne-Billancourt, France; 15 Service de Psychiatrie de L’Enfant et de L’Adolescent, Hôpital Robert Debré, APHP, Paris, France; 16 Unit of Treatment-Resistant Psychosis, Section of Psychiatry, Department of Neuroscience, Reproductive Science and Odontostomatology, University School of Medicine of Naples Federico II, Naples, Italy; 17 SCIENCES lab, Department of Psychiatry, University of Ottawa, Ottawa, Ontario, Canada; 18 Regional Centre for the Treatment of Eating Disorders and On Track: The Champlain First Episode Psychosis Program, Department of Mental Health, The Ottawa Hospital, Ontario, Canada; 19 Ottawa Hospital Research Institute Clinical Epidemiology Program University of Ottawa, Ottawa, Ontario, Canada; 20 Centre for Health Performance and Wellbeing, Anglia Ruskin University, Cambridge, United Kingdom; 21 Saint Camillus International University of Health Sciences, Rome, Italy; 22 Center for Digital Health, Medical Science Research Institute, Kyung Hee University College of Medicine, Seoul, South Korea; 23 Department of Pediatrics, Kyung Hee University Medical Center, Kyung Hee University, College of Medicine, Seoul, South Korea; 24 Université Paris Cité, Paris Saclay University, Université de Versailles Saint-Quentin-en-Yvelines, Institut National de la Santé et de la Recherche Médicale (INSERM), UMS 011 Population-based Cohorts Unit, Paris, France; 25 Université Paris Cité, Université Sorbonne Paris Nord, INSERM, Inrae, Center for Research in Epidemiology and Statistics, Paris, France

## Abstract

**Importance:**

Amid escalating mental health challenges among young individuals, intensified by the COVID-19 pandemic, analyzing postpandemic trends is critical.

**Objective:**

To examine mental health care utilization and prescription rates for children, adolescents, and young adults before and after the COVID-19 pandemic.

**Design, Setting, and Participants:**

This population-based time trend study used an interrupted time series analysis to examine mental health care and prescription patterns among the French population 25 years and younger. Aggregated data from the French national health insurance database from January 2016 to June 2023. Data were analyzed from September 2023 to February 2024.

**Main Outcomes and Measures:**

The number of individuals with at least 1 outpatient psychiatric consultation, those admitted for full-time psychiatric hospitalization, those with a suicide attempt, and those receiving psychotropic medication was computed. Data were stratified by age groups and sex. Quasi-Poisson regression modeled deseasonalized data, estimating the relative risk (RR) and 95% CI for differences in slopes before and after the pandemic.

**Results:**

This study included approximately 20 million individuals 25 years and younger (20 829 566 individuals in 2016 and 20 697 169 individuals in 2022). In 2016, the population consisted of 10 208 277 of 20 829 566 female participants (49.0%) and 6 091 959 (29.2%) aged 18 to 25 years. Proportions were similar in 2022. Significant increases in mental health care utilization were observed postpandemic compared with the prepandemic period, especially among females and young people aged 13 years and older. Outpatient psychiatric consultations increased among women (RR, 1.13; 95% CI, 1.07-1.20), individuals aged 13 to 17 years (RR, 1.15; 95% CI, 1.06-1.23), and individuals aged 18 to 25 years (RR, 1.08; 95% CI, 1.03-1.14). Hospitalizations for suicide attempt increased among women (RR, 1.14; 95% CI, 1.02-1.27) and individuals aged 18 to 25 years (RR, 1.07; 95% CI, 1.03-1.12). Regarding psychotropic medications, almost all classes, except hypnotics, increased in prescriptions between 2016 and 2022 for females, with a particularly marked rise in the postpandemic period. For men, only increases in the prescriptions of antidepressants (RR, 1.03; 95% CI, 1.01-1.06), methylphenidate (RR, 1.09; 95% CI, 1.06-1.12), and medications prescribed for alcohol use disorders (RR, 1.08; 95% CI, 1.04-1.13) were observed, and these increases were less pronounced than for women (antidepressant: RR, 1.13, 95% CI, 1.09-1.16; methylphenidate: RR, 1.15; 95% CI, 1.13-1.18; alcohol use dependence: RR, 1.12; 95% CI, 1.08-1.16). Medications reserved for severe mental health situations, such as lithium or clozapine, were prescribed more frequently starting at the age of 6 years.

**Conclusions and Relevance:**

In this study, an interrupted time-series analysis found a marked deterioration in the mental health of young women in France in the after the COVID-19 pandemic, accentuating a trend of deterioration that was already observed in the prepandemic period.

## Key Points

**Question:**

What are the trends in mental health care utilization and prescription rates among children, adolescents, and young adults in France from 2016 to 2023?

**Findings:**

In this study of approximately 20 million individuals with an interrupted time series analysis, a significant increase in mental health consultations, hospitalizations, and prescriptions for antidepressants, mood stabilizers, and antipsychotics was found among young people, particularly after the COVID-19 pandemic.

**Meaning:**

These findings suggest that the persistent rise in mental health care utilization and psychiatric medication prescriptions underscore the need for interventions that address both health care system inefficiencies and broader social determinants impacting youth mental health.

## Teaser

This study examines mental health care utilization and prescription rates for children, adolescents, and young adults after the COVID-19 pandemic.

## Introduction

In 2020, 1 in 7 adolescents globally experienced mental health issues, accounting for 13% of the global burden of morbidity in this age group.^1^ A study^2^ involving 7 519 465 children and 5 338 496 adolescents from the TriNetX Research Network revealed significant mental health distress following SARS-CoV-2 infection in youth. This was compounded by the implementation of COVID-19 pandemic-associated measures, such as school closures, social distancing guidelines, and the state of emergency status.^3^

Most of the findings have focused on the increase in anxiety and depression disorders.^4,5^ A 2020 systematic review^6^ of the psychological impact of COVID-19–related quarantine indicated that individuals experience an array of adverse effects, including anger, confusion, and posttraumatic stress symptoms. Danish youths experienced an increase in rates of psychotropic treatment and psychiatric disorder diagnoses during the COVID-19 pandemic, which was most pronounced among those aged 12 to 17 years.^7^ In Spain, the increase of suicide attempts in girls was especially prominent from September 2020 to March 2021 during the early pandemic period, where the increase reached 195%.^8^

Attentional and psychotic disorders also appear to be affected. In the US, an increase in methylphenidate prescription among young people and women was reported between 2018 and 2022. Psychotic-like experiences, including hallucinations, delusions, disorganized thinking or speech, and social withdrawal, are experienced by 40% to 66% of community adolescents and have received increased attention as some studies have suggested continuity between these experiences and psychotic disorders.^9,10,11^ In Japan^3^ and China,^12^ adolescents experienced a rapid increase in psychotic experiences at the onset of the COVID-19 pandemic.

However, data regarding the years following the initial outbreak of the pandemic remain scarce, particularly regarding whether there has been any stabilization or further deterioration in the mental health of young people in the ongoing aftermath of the pandemic. Trends indicating a deterioration in mental health of the youth were already identified before the COVID-19 pandemic.^13^ Beginning in the 2010s, these trends have primarily been concentrated among adolescents and youth.^14^ Several factors may explain why young people are particularly exposed. The increase in social inequalities manifests in many factors, including decreased family-social cohesion, poverty, and altered dietary behavior, resulting not only in increased obesity but also in a deterioration of their mental health.^15,16^ The increasingly early and prolonged exposure to screens, and more specifically, social media,^17^ increases the risk of isolation, addiction, cyberbullying, sedentariness, and impaired self-esteem.^18^ The climate crisis inducing eco-anxiety^19^ contributes to making young people more vulnerable to mental health issues with a decrease in confidence in their future.^20^ However, there is a lack of national and recent data about the association of these combined factors on the mental health of young people. Our hypothesis is that the mental health situation continued to deteriorate and that the COVID-19 pandemic amplified these existing trends. Additionally, there is a need for more detailed information on trends affecting specific genders and age groups to better describe and understand the observed phenomena and guide relevant interventions. The objective of this study, conducted from January 2016 to June 2023, was to assess whether trends in mental health care utilization and prescriptions for children, adolescents, and young adults showed signs of stabilization or further increase in the aftermath of the COVID-19 pandemic.

## Method

### Study Design, Participants, and Data Source

We conducted a population-based trend study using aggregated data from the French National Health Insurance Database (SNDS),^21^ encompassing all individuals from birth to 25 years. The SNDS captures comprehensive health care data for 98.8% of the population insured under the national health insurance system. It records all reimbursed health care services, inpatient and outpatient, regardless of the payer. The database includes anonymized demographic information and details on reimbursements for care and medications. As done in previous works, data from the SNDS are anonymized and can be reused for research purposes.^22^ According to the French law,^22^ informed consent from its participants was waived, and the study was declared to the French National Data Protection Commission. We used demographic statistics from the National Institute of Statistics and Economic Studies in France, which provides detailed census data on the French population. This study adheres to the Reporting of Studies Conducted Using Observational Routinely Collected Health Data (RECORD) Statement.^23^ The study period, from January 1, 2016, to June 30, 2023, was divided into 2 distinct periods: (1) a pre–COVID-19 pandemic period from January 2016 to February 2020, and (2) the COVID-19 pandemic and the post–COVID-19 period from March 2020 to June 2023.

### Outcome Measures

We evaluated 10 outcomes associated with mental health care utilization (number of individuals with at least 1 outpatient psychiatric consultation, those admitted for full-time psychiatric hospitalization, and individuals admitted full-time to an acute care unit for a suicide attempt identified by *International Statistical Classification of Diseases and Related Health Problems, Tenth Revision* diagnostic codes X60x to X84x, Y87.0) and psychotropic medication consumption (number of individuals who received at least 1 dispensing of various psychotropic medications, each categorized by their Anatomical Therapeutic Chemical classification, which were assessed monthly and annually) (eAppendix in Supplement 1). These outcomes were reported as rates per 1000 inhabitants and standardized for age and sex.

### Statistical Analysis

We conducted analyses for each outcome, examining them both globally and stratified by 4 age groups (ages 0 to 5 years, 6 to 12 years, 13 to 17 years, and 18 to 25 years) and by sex, using an interrupted time series (ITS) framework. Initially, we presented annual numbers and rates. We then computed monthly rates as a time series and deseasonalized the time series using a 12-month lag. Next, we used an ITS regression analyses, modeling the rate of patients per 1000 persons per month using Quasi-Poisson regression models to account for overdispersion.^24^ These models used the number of patients per month as the dependent variable and the population log (overall or across age and sex strata according to the analysis) as the offset variable. To address residual autocorrelation, we used Newey–West standard errors for coefficients, which are robust to deviations from homoscedasticity and autocorrelation (with the lag length automatically computed from the method).^25^ We estimated the relative risk (RR) and 95% CIs for the immediate change at the breakpoint (step change), and the slopes before and after the COVID-19 pandemic. We computed the RR for the difference in slopes between the 2 periods, measuring the extent to which the prepandemic slope changed postpandemic, representing the sustained change due to COVID-19. For enhanced interpretation, we computed annual RR for slopes in both periods by either multiplying coefficients by 12 or exponentiating monthly RR by 12. Lastly, we calculated the relative difference of 2016 to 2022 rates for various medications across age groups and sexes.

We conducted analyses using R version 4.1.2 (R Project for Statistical Computing), using the glm() and ts() functions from the stats package for deseasonalization and modeling, and the sandwich() package for computing and using standard errors via the Newey–West method. All tests were 2-sided, with a significance level set at *P* < .05. Data were analyzed from September 2023 to February 2024.

## Results

This study included approximately 20 million individuals 25 years and younger (20 829 566 individuals in 2016 and 20 697 169 individuals in 2022). In 2016, the population consisted of 10 208 277 of 20 829 566 female participants (49.0%) and 6 091 959 (29.2%) aged 18 to 25 years. Proportions were similar in 2022. Comparing trends before and after the pandemic, we observed a significant increase in the relative monthly rate of change in outpatient psychiatric consultations for females (RR, 13%, 95% CI, 7%-20%), ages 13 to 17 years (RR, 15%; 95% CI, 6%-23%), and ages 18 to 25 years (RR, 8%; 95% CI, 3%-14%). Psychiatric full-time hospitalizations increased in females (RR, 9%; 95% CI, 2%-18%). In males, a smaller increase was also observed (RR, 5%; 95% CI, 0%-9%), but this followed a slight decrease in the prepandemic period. The hospitalizations for suicide attempt increased among females (RR, 14%; 95% CI, 2%-27%) and individuals aged 18 to 25 years (RR, 7%; 95% CI, 3%-12%).

**Figure 1.**
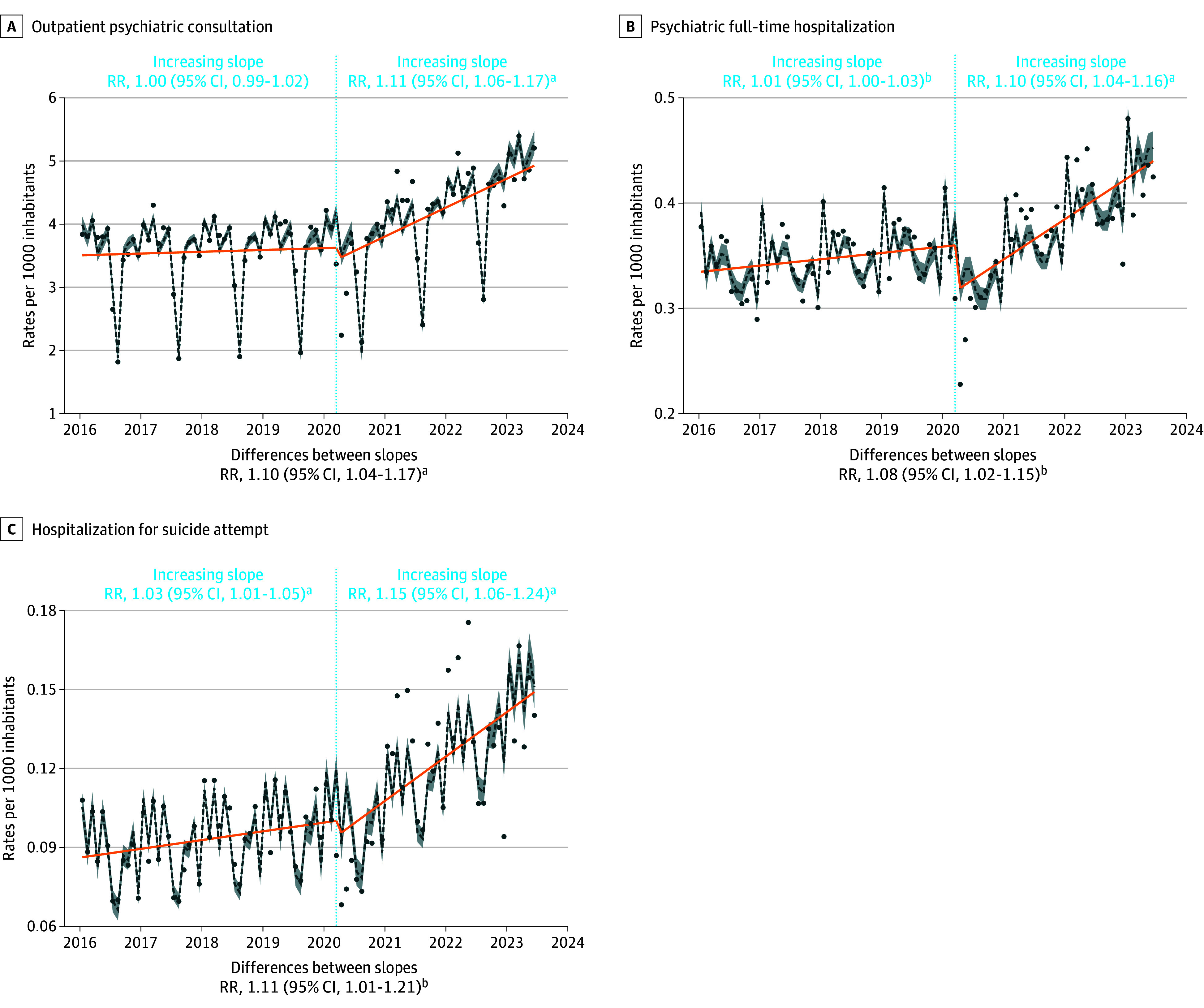
Interrupted Time Series Analysis of Changes in Mental Health Care Utilization Before and After the Beginning of the COVID-19 Pandemic With Annual Relative Risk (RR) ^a^*P* < .001. ^b^*P* < .05. The reported RR are annual and can be interpreted as follows, using full-time psychiatric hospitalization as an example: before March 2020, the annual rate of these hospitalizations increased significantly by 1% (RR = 1.01). After March 2020, this rate increased annually by 10% (RR = 1.10). Consequently, the annual trend was considered to have significantly accelerated by 8% (RR for the difference in slopes = 1.08) after March 2020 compared with before. The dotted lines are Quasi-Poisson estimates with shading indicating 95% CIs. Dots are actual observations. Orange line indicates the annual trend. The rates are expressed per 1000 inhabitants.

**Figure 2.**
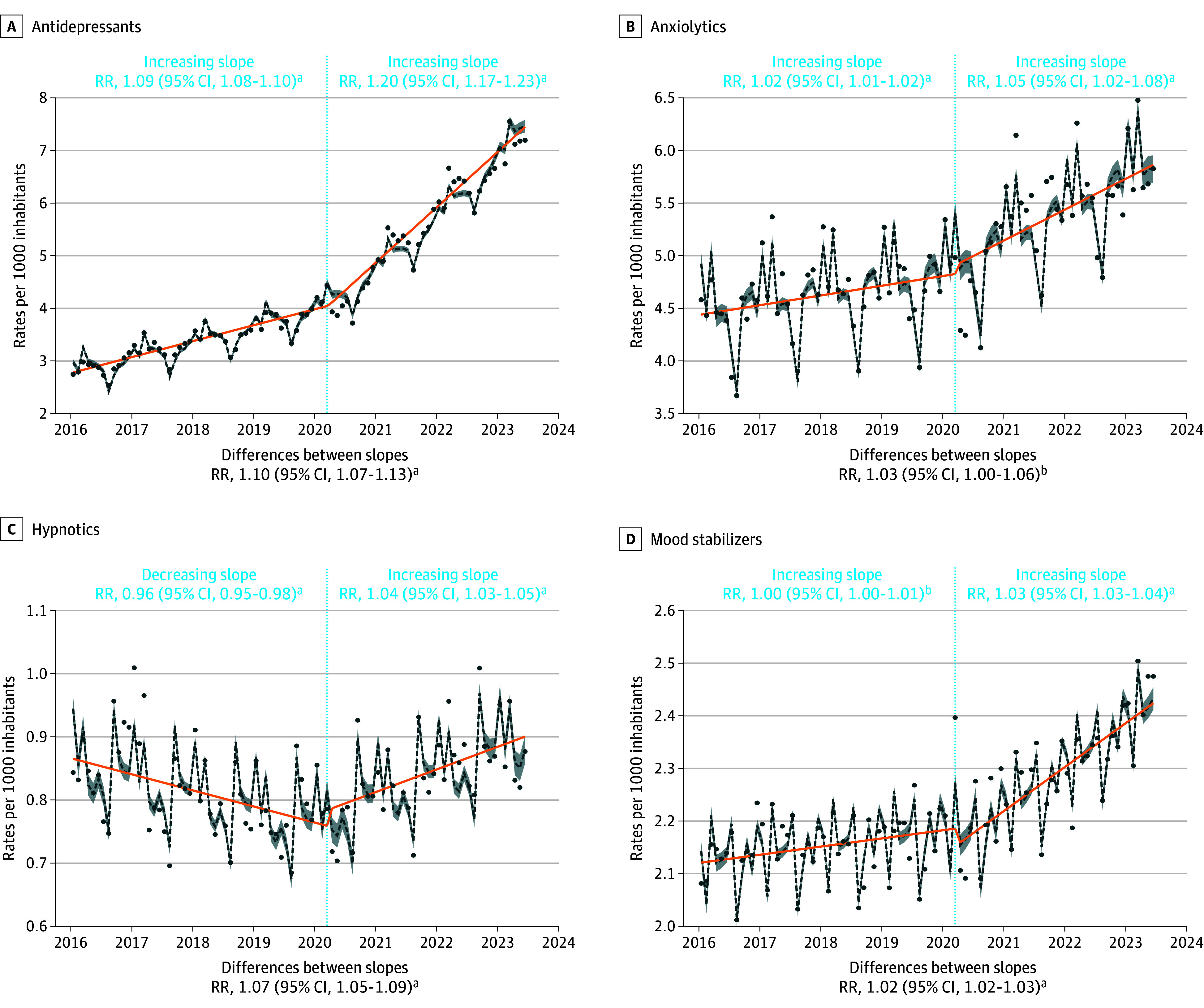
Interrupted Time Series Analysis of Changes in Prescriptions of Antidepressants, Anxiolytics, Hypnotics, and Mood Stabilizers Before and After the Beginning of the COVID-19 Pandemic With Annual Relative Risk (RR) ^a^*P* < .001. ^b^*P* < .05. The reported RR are annual and can be interpreted as follows, using antidepressants prescriptions as an example: before March 2020, the annual rate of these prescriptions increased significantly by 9% (RR = 1.09). After March 2020, this rate increased annually by 20% (RR = 1.20). Consequently, the annual trend was considered to have significantly accelerated by 10% (RR for the difference in slopes = 1.10) after March 2020 compared with before. The dotted lines are quasi Poisson estimates with shading indicated 95% CIs. Dots are actual observations. Orange line indicates the annual trend. The rates are expressed per 1000 inhabitants.

**Figure 3.**
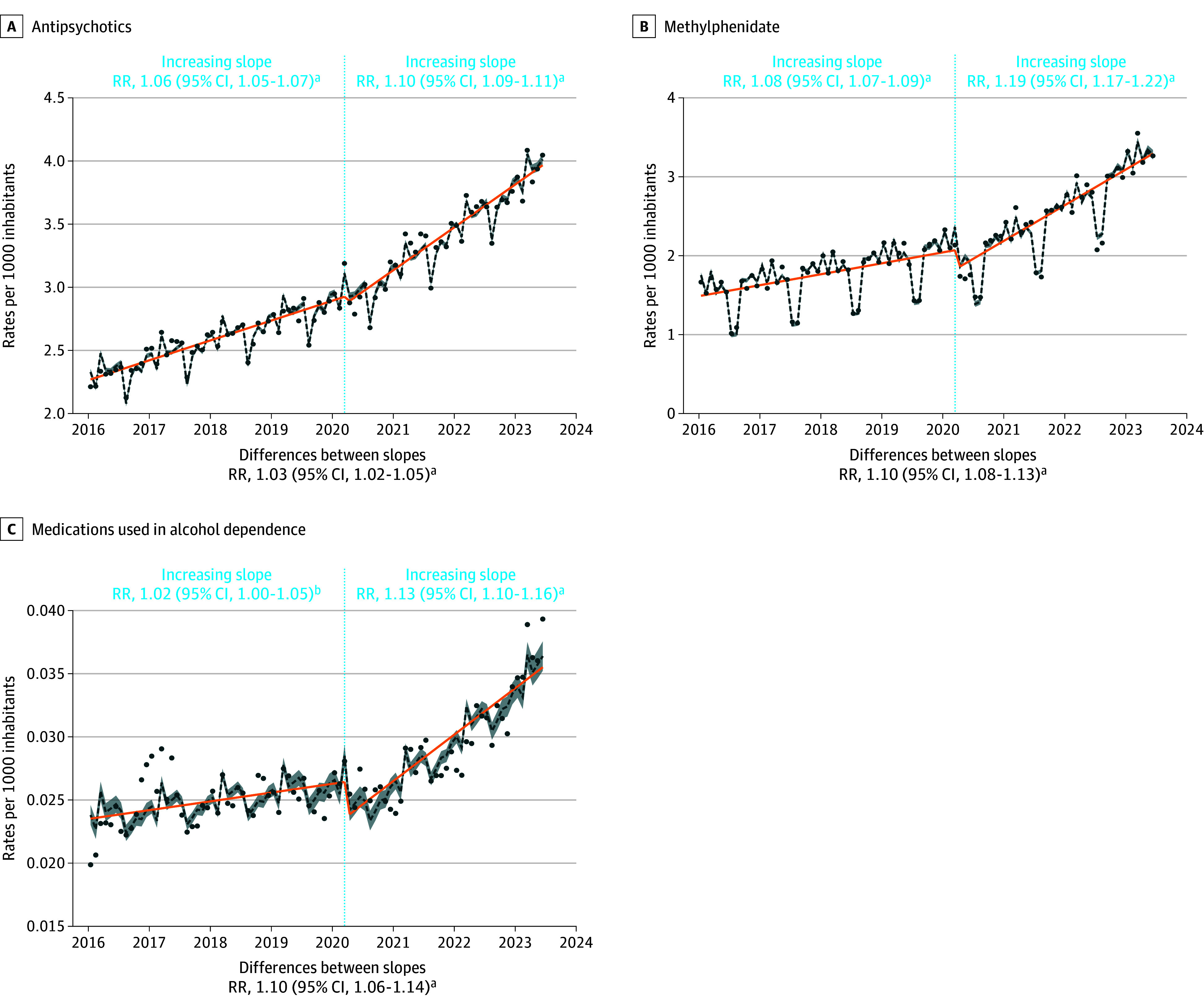
Interrupted Time Series Analysis of Changes in Prescriptions of Antipsychotics, Methylphenidate, and Medications for Alcohol Dependence Before and After the Beginning of the COVID-19 Pandemic With Annual Relative Risk (RR) ^a^*P* < .001. ^b^*P* < .05. The reported RR are annual and can be interpreted as follows, using antipsychotics prescriptions as an example: before March 2020, the annual rate of these prescriptions increased significantly by 6% (RR = 1.06). After March 2020, this rate increased annually by 10% (RR = 1.10). Consequently, the annual trend was considered to have significantly accelerated by 3% (RR for the difference in slopes = 1.03) after March 2020 compared with before. The dotted lines are quasi-Poisson estimates with shading indicating 95% CI. Dots are actual observations. Orange line indicates the annual trend. The rates are expressed per 1000 inhabitants.

The largest increases were observed for the prescription of antidepressants (females: RR, 13%; 95% CI, 9%-16%; males: RR, 3%; 95% CI, 1%-6%) and methylphenidate (females: RR, 15%; 95% CI, 13%-18%; males: RR, 9%; 95% CI, 6%-12%) in both sexes but more pronounced in females. For methylphenidate, the relative difference is less pronounced for males than for females but the absolute difference is more significant for males, who received much more methylphenidate prescriptions than females in the prepandemic period. The prescription of anxiolytics increased significantly among females (RR, 5%; 95% CI, 1%-9%), ages 0 to 5 years (RR, 17%; 95% CI, 8%-25%), and ages 6 to 12 years (RR, 8%; 95% CI, 4%-11%). The prescription of anxiolytics was already increasing among females in the prepandemic period, whereas it was decreasing among children aged 0 to 5 years and 6 to 12 years. The prescription of hypnotics increased significantly among ages 13 to 17 years (RR, 9%; 95% CI, 4%-15%) and ages 18 to 25 years (RR, 8%; 95% CI, 6%-10%). This followed a significant decrease in prescriptions in these groups during the prepandemic period. The prescription of mood stabilizers (RR, 5%; 95% CI, 4%-6%) and antipsychotics (RR, 9%; 95% CI, 7%-11%) increased among females with an acceleration in the postpandemic period. The prescription of medications used in alcohol dependence increased significantly among ages 18 to 25 years (RR, 10%; 95% CI, 6%-15%) (Figures 1, 2, and 3; eFigures 1 and 2 and eTables 1 and 2 in Supplement 1).

The details for the relevant medications are presented in the Table; eTable 3 in Supplement 1. The most substantial increases were observed for lithium and lamotrigine as early as age 6 years (relative difference of 2016 to 2022 ratios: lithium: ages 6 to 12 years, 87%; ages 13 to 17 years, 137%; ages 18 to 25 years, 149%; lamotrigine: ages 6 to 12 years, 6%; ages 13 to 17 years, 20%; ages 18 to 25 years, 67%), chlorpromazine (150%), quetiapine (217%), and aripiprazole (177%) in all age groups especially in females, clozapine in ages 6 to 12 years (219%) and ages 13 to 17 years (157%), and fluoxetine (162%), sertraline (214%), mirtazapine (123%), and venlafaxine (101%) in both sexes and all age groups.

**Table.** Change in Prescription Rates Between 2016 and 2022 by Medication^a^

Medication	Change by subgroup, %							
	All	Men	Women		Age group, y			
					0-5 y	6-12 y	13-17 y	18-25 y
Mood stabilizers								
Carbamazepine	3	−2		8	21	−1	−8	3
Valproic acid	−17	−7		−33	−28	−22	−21	−8
Lamotrigine	48	29		60	−1	6	20	67
Lithium	155	89		208	−5	87	137	149
Antipsychotics								
Chlorpromazine	119	81		150	16	90	201	89
Levomepromazine	52	25		105	−39	39	85	35
Cyamemazine	77	31		155	32	45	82	72
Periciazine	−20	−22		−14	−37	−13	−20	−24
Haloperidol	0	−3		4	−44	−14	−1	−3
Pipamperone	−32	−32		−31	−25	−37	−47	−24
Zuclopenthixol	10	1		39	120	−29	−20	16
Loxapine	80	45		150	88	90	77	71
Clozapine	99	81		138	NA	219	157	84
Olanzapine	47	19		94	−18	37	61	38
Quetiapine	142	63		217	70	98	233	120
Sulpiride	−37	−41		−34	−47	−57	−48	−34
Tiapride	−31	−37		−19	−72	−39	−47	−25
Amisulpride	24	2		61	−32	42	4	20
Risperidone	28	19		54	46	38	16	21
Aripiprazole	114	71		177	189	182	119	91
Paliperidone	33	34		28	10	78	0	29
Anxiolytics								
Diazepam	31	24		37	−37	−25	26	64
Oxazepam	135	91		164	53	63	120	126
Potassium clorazepate	−6	−19		6	−39	−35	−21	−6
Lorazepam	85	58		104	−5	42	86	77
Bromazepam	−9	−14		−6	−35	−26	−22	−11
Clobazam	−2	−2		−3	−20	−10	−8	−1
Prazepam	14	0		21	−26	−30	5	12
Alprazolam	45	29		53	−3	7	33	40
Clotiazepam	14	2		20	2	−14	8	10
Hydroxyzine	15	3		24	−9	13	24	16
Buspirone	37	20		46	10	68	44	28
Hypnotics, excluding melatonin								
Lormetazepam	55	34		71	3	43	24	51
Midazolam	80	82		77	89	71	79	176
Loprazolam	−3	−10		3	27	−42	−34	−4
Zoplicone	60	46		70	38	28	17	57
Zolpidem	−83	−83		−83	−78	−75	−85	−84
Antidepressants								
Clomipramine	−6	−27		19	−60	−56	−18	21
Amitriptyline	25	8		34	−28	−12	5	37
Fluoxetine	162	111		187	49	143	230	126
Citalopram	−36	−46		−31	−67	−45	−37	−39
Paroxetine	94	60		116	54	39	83	87
Sertraline	214	133		269	101	121	193	216
Escitalopram	18	1		27	−22	−10	12	14
Mianserine	57	34		72	12	−2	37	54
Mirtazapine	123	87		152	34	52	114	114
Venlafaxine	102	70		121	2	18	103	96
Duloxetine	40	13		54	10	−5	26	36
Agomelatine	−82	−81		−82	−100	−90	−67	−84
Alcohol dependence medications								
Acamprosate	42	33		69	−76	69	−16	38
Naltrexone	43	19		86	−18	−17	−24	43
Nalmefene	−22	−33		8	−53	−56	−43	−24

Abbreviation: NA, not applicable (division by 0).

^a^ Detailed data are available in eTable 3 in Supplement 1.

## Discussion

Our results revealed substantial differences in trends before and after the COVID-19 pandemic by sex and age. For males, mental health care utilization (eg, outpatient consultations, psychiatric hospitalizations, and hospitalizations for suicide attempt) and almost all classes of psychotropic medication prescriptions have increased between 2016 and 2023, with a marked increase in the trend from 2020 to 2023. The increase was less pronounced for males than for females, which in some cases followed a decrease during the prepandemic period. Although a general increase in the use of psychotropic medications affected all age groups, it was observed that in those older than age 13 years, the postpandemic increase was an acceleration or intensification of the increase observed in the prepandemic period. Meanwhile, in younger children, the postpandemic increase occured following a prepandemic decrease in most cases and therefore it was difficult to conclude the interpretation for this age group. However, for certain medications reserved for severe cases, such as lithium and clozapine, an increase of prescriptions was noted from the age of 6 years. The increases in prescriptions of mood stabilizers, antipsychotics, and anxiolytics for females followed increases already noted before the COVID-19 pandemic, but with an acceleration in the postpandemic period, while males did not seem to be affected by these trends for these classes of psychotropics.

After the COVID-19 pandemic, a significant change in health care utilization has been observed among females and from the age of 13 years onwards in our results. This trend aligned with studies indicating that COVID-19 infection and lockdowns have had biological and societal impacts on the mental health of the youth^26,27^ with the outcomes of a recent meta-analysis, which identified female sex as a risk factor for experiencing mental health complications during epidemics, including the COVID-19 pandemic.^27^ People with preexisting mental health symptoms were also identified as being at greater risk of developing post–COVID complications,^28^ which can also explain in part our results for women. There may be other explanations for the observed trends. The role of social media in the increased suicidal behavior in young girls was currently questioned.^29,30,31^ Social media has become a primary forum for interpersonal engagement in adolescence—a developmental period when social contact was rapidly rising and becoming increasingly important to well-being—making this an area of significant potential influence and importance.^32^ Another key feature of social media was the amount of time adolescents spend engaged with it and the fact that it makes social contact available almost without limits. These features and secular trends strongly suggest that social media should be a key target of interest for the trends reported in suicidality in youth.^31^ Compared with boys, girls’ social media use may be more frequent^33^ more exposed to cyberbullying^34^ and likely to result in interpersonal stress, a common factor associated with suicide attempts^35^ and depression.^33^ Girls with depression elicit more negative responses from peers on social media compared with boys with depression.^36^ Longitudinal latent profiles of aggressive and impulsive behaviors were associated with increased suicide attempts in females but not in males,^37^ which may explain the sex differences in our results. Suicide attempt admission was associated with the long-term risk of eating disorder hospitalization in adolescent girls.^38^ Other factors, such as family cohesion, a parent’s loss of employment during the COVID-19 pandemic, poverty, and additional elements, may also be considered.

The overall increase in prescriptions of antidepressants (more pronounced in women and for all age groups from the age of 6 years) suggested a rise in severe anxiety-depressive disorders. For the specific case of methylphenidate (whose prescription was highly restricted in France and specific to attention deficit/hyperactivity disorder [ADHD] diagnosis, having been tightly regulated since 2002), the sharp increase in prescriptions among those aged 18 to 25 years after the pandemic may be explained by the increasing recognition of adult ADHD and through prolonged prescriptions of methylphenidate into adulthood.^39^ The ADHD diagnosis among children and adolescents in France increased steadily by 96% between 2011 and 2019, and the number of children diagnosed with ADHD and hospitalized in France increased by 167% among those aged 12 to 17 years.^40^ However, we cannot rule out the possibility that attention disorders were increasing across all population segments and that this phenomenon had been accelerated since the COVID-19 crisis.

Females have received more prescriptions for mood stabilizers, antipsychotics, and anxiolytics from 2020 to 2023, accelerating a trend that was already present before the COVID-19 pandemic. The increase in lithium prescriptions starting from 6 years old is particularly striking. This medication was licensed for bipolar disorder and suggested an increase in the incidence of bipolar disorders in children, adolescents, and young adults following the COVID-19 pandemic. We have no evidence to suggest that the screening for bipolar disorder in children and adolescents had significantly improved during the 2020 to 2023 period compared with the 2016 to 2019 period. On the contrary, the disorganization of care and the sharp decrease in the number of mental health professionals^41^ should have rather led to a decrease or stagnation in prescriptions. In our results, the increase in lithium and lamotrigine prescriptions was part of an overall rise in mood stabilizer prescriptions, indicating that these increases were not solely explained by the decrease in valproate or other mood regulator prescriptions. This was consistent with the global increase in the incidence of bipolar disorder among adolescents and young adults.^42^ Adverse stressful life events have been identified as the most prominent environmental risk factor for triggering bipolar disorder in adolescence.^43^ Thus, the phenomenon existed before the COVID-19 pandemic but appeared to have been accelerated over the past 3 years.

Among antipsychotics, aripiprazole and quetiapine may be prescribed in the first episodes of psychosis or during manic or mixed episodes, as well as for irritability and impulsive aggression. However, the increase in prescriptions of clozapine from the age of 6 years suggests an early onset of first psychotic episodes, with suicidal ideation being the main indication for clozapine among youth, which otherwise is used not much due to adverse effect burden and monitoring requirements.^44^ This finding may indicate a worsening in the severity of early schizophrenia among young people following the COVID-19 pandemic, which had not returned to previous baseline metrics yet. The increase in prescriptions observed for chlorpromazine (a first-generation antipsychotic) in all groups, but particularly noticeable from the age of 6 years, was likely explained by its potential benefits in protecting against severe COVID-19 infection.^45^ This medication is also used for calming purposes, similar to loxapine and cyamemazine, which have also seen increased prescriptions in our results. These medications may be more commonly used in children and adolescents because benzodiazepines were used more cautiously in this population due to the potential risk of disinhibition and voluntary medication overdose in the case of suicidal thoughts.^46^ This is a possible explanation for the observed increase in the prescription of anxiolytics, particularly among children aged 6 to 12 years in our results.

### Strengths and Limitations

This study provides the most updated data, covering up to 2023, more than 20 million individuals younger than 25 years old and 10 outcomes associated with mental health care utilization and psychotropic medication consumption. We used ITS analysis, which accounts for both secular trends and seasonal variations, allowing for interpretable and understandable findings. We also used precise and correct estimations, correcting for overdispersion, heteroscedascticity, and residual autocorrelation, resulting in more accurate results.

This study has limitations. Our study cannot conclude whether the increase in the use of services and prescriptions reflects different attitudes of individuals and prescribers or a true deterioration in mental health. However, the medical demographic crisis in France, with a 34% decrease in the number of child psychiatrists between 2010 and 2022,^41^ making access to mental health care for children and adolescents increasingly difficult, tends to indicate an alarming deterioration in mental health of young women in France. The absolute number of individuals with at least 1 prescription for each class of psychotropic medication is increasing, which does not support the hypothesis of transfers of prescriptions from certain molecules to others. Additionally, our results are likely underestimated. Only a small proportion of mental disorders receive pharmacological treatment, especially among children and adolescents.^47^ Similarly, the coding for suicide attempt may be underreported. There are also significant issues with access to child and adolescent psychiatry services in France. Finally, future studies should also explore the intensity of service use in addition to the number of unique individuals affected.

## Conclusions

These findings suggest that mental health care utilization and psychotropic medication prescriptions increased among young people during the COVID-19 pandemic, with many trends persisting postpandemic. These results suggest a deterioration in mental health, likely due to direct and indirect COVID-19 factors, as well as preexisting factors that require further evaluation to guide targeted interventions.

## Notes

Supplement 1. **eAppendix.** Anatomical Therapeutic Chemical (ATC) Classification **eTable 1.** Mental Health Care and Prescription Trends Before and After the Beginning of the COVID-19 Pandemic **eTable 2.** Relative-Risk (RR) Difference Between Annual Trends After vs Before the COVID-19 Pandemic, and Immediate Effect Estimation **eTable 3.** Change in Prescription Rates Between 2016 and 2022 by Drug **eFigure 1.** Sex-Specific Trends in Mental Health Care and Prescriptions Before and After the Beginning of the COVID-19 Pandemic With Annual Relative Risk **eFigure 2.** Age-Specific Trends in Mental Health Care and Prescriptions Before and After the Beginning of the COVID-19 Pandemic With Annual Relative Risk

Supplement 2.
Data Sharing Statement
