# Supplementary material for: Mental Health Care Utilization and Prescription Rates Among Children, Adolescents, and Young Adults in France
Source: JAMA Netw Open. 2025 Jan 7;8(1):e2452789. doi: 10.1001/jamanetworkopen.2024.52789 (PMC11707635; doi:10.1001/jamanetworkopen.2024.52789)
Supplement: Supplement 1. — eAppendix. Anatomical Therapeutic Chemical (ATC) Classification eTable 1. Mental Health Care and Prescription Trends Before and After the Beginning of the COVID-19 Pandemic eTable 2. Relative-Risk (RR) Difference Between Annual Trends After vs Before the COVID-19 Pandemic, and Immediate Effect Estimation eTable 3. Change in Prescription Rates Between 2016 and 2022 by Drug eFigure 1. Sex-Specific Trends in Mental Health Care and Prescriptions Before and After the Beginning of the COVID-19 Pandemic With Annual Relative Risk eFigure 2. Age-Specific Trends in Mental Health Care and Prescriptions Before and After the Beginning of the COVID-19 Pandemic With Annual Relative Risk [file jamanetwopen-e2452789-s001.pdf]

Supplemental Online Content

Fond G, Pauly V, Brousse Y, et al. Mental Health Care Utilization and Prescription Rates Among Children, Adolescents, and Young Adults in France. *JAMA Netw Open*. 2024;7(12):e2452789. doi:10.1001/jamanetworkopen.2024.52789

eAppendix. Anatomical Therapeutic Chemical (ATC) Classification
eTable 1. Mental Health Care and Prescription Trends Before and After the Beginning of the COVID-19 Pandemic
eTable 2. Relative-Risk (RR) Difference Between Annual Trends After vs Before the COVID-19 Pandemic, and Immediate Effect Estimation
eTable 3. Change in Prescription Rates Between 2016 and 2022 by Drug
eFigure 1. Sex-Specific Trends in Mental Health Care and Prescriptions Before and After the Beginning of the COVID-19 Pandemic With Annual Relative Risk
eFigure 2. Age-Specific Trends in Mental Health Care and Prescriptions Before and After the Beginning of the COVID-19 Pandemic With Annual Relative Risk

This supplemental material has been provided by the authors to give readers additional information about their work.

© 2024 Fond G et al. *JAMA Network Open*.

eAppendix. Anatomical Therapeutic Chemical (ATC) Classification

This included antidepressants (ATC N06A, excluding bupropion N06AX12), anxiolytics (ATC N05B), hypnotics and sedatives (ATC N05C, excluding melatonin, which was only reimbursed starting from 2020), mood stabilizers (including lithium ATC N05AN, carbamazepine ATC N03AF01, valproic acid ATC N03AG01, valpromide ATC N03AG02, and lamotrigine ATC N03AX09), antipsychotics (ATC N05A, excluding lithium ATC N05AN, veralipride ATC N05AL06, and Neuriplege® with French CIP code 3400932896332), methylphenidate (ATC N06BA04), and medications used for alcohol dependence (ATC N07BB).

© 2024 Fond G et al. *JAMA Network Open*.

eTable 1. Mental Health Care and Prescription Trends Before and After the Beginning of the COVID-19 Pandemic

	2016	2017	2018	2019	2020	2021	2022
Outpatient psychiatric consultation, N (rates per 1000 inhabitants)	267,670 (12.85)	272,081 (13.09)	270,410 (13.01)	275,713 (13.30)	265,070 (12.79)	299,497 (14.46)	315,272 (15.23)
Females	128,595 (12.60)	132,839 (13.04)	132,756 (13.05)	136,113 (13.42)	135,583 (13.38)	162,608 (16.07)	176,393 (17.46)
Males	139,080 (13.09)	139,251 (13.13)	137,656 (12.98)	139,608 (13.18)	129,493 (12.22)	136,892 (12.92)	138,881 (13.11)
0 – 5 years	29,089 (6.19)	27,417 (5.92)	25,105 (5.52)	23,891 (5.33)	20,327 (4.62)	19,720 (4.55)	17,718 (4.14)
6 – 12 years	82,956 (14.15)	78,319 (13.32)	75,400 (12.80)	75,063 (12.81)	66,688 (11.39)	67,516 (11.61)	64,329 (11.15)
13 – 17 years	61,560 (14.74)	60,114 (14.31)	60,535 (14.46)	61,432 (14.66)	59,388 (14.07)	70,084 (16.42)	75,228 (17.46)
18 – 25 years	102,910 (16.89)	114,947 (18.91)	118,312 (19.23)	124,374 (20.05)	127,209 (20.32)	152,063 (24.16)	168,543 (26.58)
Psychiatric full-time hospitalization, N (rates per 1000 inhabitants)	49,334 (2.37)	50,987 (2.45)	52,949 (2.55)	53,898 (2.60)	48,605 (2.34)	57,292 (2.77)	60,982 (2.95)
Females	22,823 (2.24)	24,227 (2.38)	25,583 (2.52)	26,625 (2.63)	25,563 (2.52)	32,819 (3.24)	36,149 (3.58)
Males	26,515 (2.50)	26,762 (2.52)	27,368 (2.58)	27,274 (2.57)	23,043 (2.17)	24,477 (2.31)	24,835 (2.34)
0 – 5 years	367 (0.08)	347 (0.07)	290 (0.06)	266 (0.06)	148 (0.03)	151 (0.03)	182 (0.04)
6 – 12 years	4,232 (0.72)	4,221 (0.72)	4,125 (0.70)	4,179 (0.71)	2,942 (0.50)	3,369 (0.58)	3,799 (0.66)
13 – 17 years	14,412 (3.45)	15,102 (3.60)	15,726 (3.76)	15,527 (3.70)	14,212 (3.37)	17,540 (4.11)	18,500 (4.29)
18 – 25 years	30,948 (5.08)	32,024 (5.27)	33,559 (5.45)	34,616 (5.58)	32,036 (5.12)	37,054 (5.89)	39,301 (6.20)
Suicide attempt, N (rates per 1000 inhabitants)	19,512 (0.94)	19,920 (0.96)	21,621 (1.04)	21,793 (1.05)	19,354 (0.93)	26,219 (1.27)	27,921 (1.35)
Females	13,836 (1.36)	14,214 (1.39)	15,293 (1.50)	15,438 (1.52)	13,641 (1.35)	19,906 (1.97)	21,611 (2.14)
Males	5,665 (0.53)	5,700 (0.54)	6,319 (0.60)	6,337 (0.60)	5,696 (0.54)	6,300 (0.59)	6,300 (0.59)
0 – 5 years	178 (0.04)	152 (0.03)	196 (0.04)	172 (0.04)	172 (0.04)	167 (0.04)	169 (0.04)
6 – 12 years	800 (0.14)	885 (0.15)	991 (0.17)	1,049 (0.18)	930 (0.16)	1,431 (0.25)	1,245 (0.22)
13 – 17 years	9,165 (2.19)	9,453 (2.25)	10,342 (2.47)	9,915 (2.37)	8,503 (2.01)	13,250 (3.10)	14,122 (3.28)
18 – 25 years	9,433 (1.55)	9,493 (1.56)	10,176 (1.65)	10,761 (1.73)	9,814 (1.57)	11,517 (1.83)	12,531 (1.98)
Antidepressants, N (rates per 1000 inhabitants)	205,948 (9.89)	230,678 (11.09)	238,912 (11.50)	250,718 (12.09)	261,529 (12.61)	322,468 (15.56)	368,822 (17.82)
Females	129,890 (12.72)	146,072 (14.34)	151,385 (14.88)	159,890 (15.77)	170,500 (16.83)	218,039 (21.55)	254,949 (25.23)
Males	76,059 (7.16)	84,607 (7.98)	87,531 (8.25)	90,831 (8.57)	91,037 (8.59)	104,435 (9.85)	113,877 (10.75)
0 – 5 years	2,480 (0.53)	2,315 (0.50)	2,161 (0.47)	2,036 (0.45)	1,785 (0.41)	1,935 (0.45)	2,219 (0.52)
6 – 12 years	10,472 (1.79)	10,214 (1.74)	10,008 (1.70)	9,986 (1.70)	9,813 (1.68)	11,296 (1.94)	12,197 (2.11)
13 – 17 years	39,829 (9.53)	41,110 (9.79)	44,002 (10.51)	46,581 (11.11)	49,389 (11.70)	67,085 (15.72)	80,279 (18.63)
18 – 25 years	158,051 (25.94)	182,110 (29.96)	188,494 (30.63)	198,425 (31.98)	207,522 (33.15)	251,397 (39.94)	285,419 (45.02)
Anxiolytics, N (rates per 1000 inhabitants)	651,766 (31.29)	679,155 (32.66)	678,379 (32.65)	675,703 (32.59)	653,714 (31.53)	727,072 (35.09)	706,149 (34.12)

© 2024 Fond G et al. *JAMA Network Open.*

	2016	2017	2018	2019	2020	2021	2022
Females	399,627 (39.15)	418,470 (41.07)	417,192 (41.02)	415,242 (40.94)	407,294 (40.20)	464,374 (45.89)	449,255 (44.47)
Males	252,142 (23.74)	260,688 (24.59)	261,191 (24.62)	260,464 (24.59)	246,421 (23.25)	262,701 (24.79)	256,896 (24.25)
0 – 5 years	56,610 (12.06)	50,071 (10.81)	48,197 (10.59)	45,811 (10.22)	33,945 (7.72)	38,783 (8.94)	43,281 (10.12)
6 – 12 years	62,692 (10.69)	60,582 (10.30)	60,576 (10.29)	60,008 (10.24)	56,507 (9.65)	63,474 (10.91)	65,578 (11.37)
13 – 17 years	129,517 (31.00)	127,710 (30.41)	127,638 (30.49)	125,015 (29.83)	120,549 (28.57)	143,649 (33.65)	134,475 (31.21)
18 – 25 years	409,148 (67.16)	447,076 (73.55)	448,476 (72.88)	451,467 (72.77)	449,567 (71.82)	489,301 (77.73)	470,996 (74.28)
Hypnotics, N (rates per 1000 inhabitants)	117,514 (5.64)	112,968 (5.43)	108,217 (5.21)	104,828 (5.06)	101,982 (4.92)	105,877 (5.11)	114,296 (5.52)
Females	68,212 (6.68)	64,156 (6.30)	60,327 (5.93)	57,850 (5.70)	56,549 (5.58)	60,444 (5.97)	64,974 (6.43)
Males	49,303 (4.64)	48,813 (4.60)	47,891 (4.51)	46,979 (4.43)	45,434 (4.29)	45,433 (4.29)	49,322 (4.66)
0 – 5 years	7,911 (1.68)	8,853 (1.91)	9,716 (2.14)	10,238 (2.28)	9,453 (2.15)	9,982 (2.30)	13,016 (3.04)
6 – 12 years	8,627 (1.47)	9,825 (1.67)	11,098 (1.88)	11,559 (1.97)	12,332 (2.11)	11,482 (1.97)	13,633 (2.36)
13 – 17 years	13,897 (3.33)	12,600 (3.00)	12,649 (3.02)	12,337 (2.94)	12,214 (2.89)	12,750 (2.99)	14,049 (3.26)
18 – 25 years	88,008 (14.45)	82,520 (13.58)	75,689 (12.30)	71,613 (11.54)	68,973 (11.02)	72,552 (11.53)	74,614 (11.77)
Mood stabilizers, N (rates per 1000 inhabitants)	77,174 (3.71)	78,607 (3.78)	77,894 (3.75)	78,164 (3.77)	77,730 (3.75)	80,710 (3.90)	84,175 (4.07)
Females	35,051 (3.43)	35,206 (3.46)	34,829 (3.42)	35,368 (3.49)	35,872 (3.54)	38,333 (3.79)	41,486 (4.11)
Males	42,126 (3.97)	43,404 (4.09)	43,072 (4.06)	42,798 (4.04)	41,860 (3.95)	42,378 (4.00)	42,689 (4.03)
0 – 5 years	10,090 (2.15)	8,979 (1.94)	8,387 (1.84)	7,885 (1.76)	7,297 (1.66)	7,014 (1.62)	7,213 (1.69)
6 – 12 years	17,243 (2.94)	16,409 (2.79)	16,021 (2.72)	15,863 (2.71)	15,379 (2.63)	14,898 (2.56)	14,711 (2.55)
13 – 17 years	18,059 (4.32)	17,499 (4.17)	17,199 (4.11)	17,030 (4.06)	16,754 (3.97)	17,200 (4.03)	17,878 (4.15)
18 – 25 years	37,130 (6.09)	41,125 (6.77)	41,597 (6.76)	42,674 (6.88)	43,553 (6.96)	46,685 (7.42)	49,656 (7.83)
Antipsychotics, N (rates per 1000 inhabitants)	118,633 (5.70)	129,801 (6.24)	135,365 (6.51)	141,563 (6.83)	145,809 (7.03)	164,849 (7.96)	180,096 (8.70)
Females	39,885 (3.91)	44,438 (4.36)	47,576 (4.68)	50,779 (5.01)	55,105 (5.44)	69,168 (6.83)	81,011 (8.02)
Males	78,751 (7.41)	85,368 (8.05)	87,793 (8.28)	90,788 (8.57)	90,706 (8.56)	95,683 (9.03)	99,088 (9.35)
0 – 5 years	2,435 (0.52)	2,452 (0.53)	2,463 (0.54)	2,444 (0.55)	2,440 (0.55)	2,750 (0.63)	2,831 (0.66)
6 – 12 years	22,286 (3.80)	22,984 (3.91)	24,078 (4.09)	25,608 (4.37)	26,274 (4.49)	28,910 (4.97)	30,486 (5.28)
13 – 17 years	36,072 (8.63)	37,051 (8.82)	38,943 (9.30)	40,760 (9.72)	41,815 (9.91)	49,842 (11.68)	55,306 (12.83)
18 – 25 years	64,265 (10.55)	73,917 (12.16)	77,011 (12.51)	80,159 (12.92)	83,014 (13.26)	92,076 (14.63)	100,871 (15.91)
Methylphenidate, N (rates per 1000 inhabitants)	64,893 (3.12)	70,605 (3.40)	75,989 (3.66)	82,614 (3.98)	85,653 (4.13)	98,354 (4.75)	116,123 (5.61)
Females	12,840 (1.26)	14,197 (1.39)	15,491 (1.52)	17,139 (1.69)	17,987 (1.78)	21,776 (2.15)	27,975 (2.77)
Males	52,056 (4.90)	56,413 (5.32)	60,500 (5.70)	65,478 (6.18)	67,669 (6.38)	76,583 (7.23)	88,149 (8.32)
0 – 5 years	909 (0.19)	1,004 (0.22)	985 (0.22)	1,074 (0.24)	1,016 (0.23)	1,225 (0.28)	1,342 (0.31)

© 2024 Fond G et al. *JAMA Network Open*.

	2016	2017	2018	2019	2020	2021	2022
6 – 12 years	39,647 (6.76)	42,663 (7.25)	45,401 (7.71)	49,319 (8.42)	50,692 (8.66)	58,171 (10.00)	66,661 (11.55)
13 – 17 years	23,728 (5.68)	25,773 (6.14)	28,069 (6.70)	30,457 (7.27)	31,475 (7.46)	35,258 (8.26)	40,988 (9.51)
18 – 25 years	6,163 (1.01)	7,205 (1.19)	8,074 (1.31)	8,931 (1.44)	9,806 (1.57)	12,166 (1.93)	16,793 (2.65)
Drugs used in alcohol dependence, N (rates per 1000 inhabitants)	2,754 (0.13)	2,987 (0.14)	2,923 (0.14)	2,946 (0.14)	2,859 (0.14)	3,019 (0.15)	3,291 (0.16)
Females	754 (0.07)	854 (0.08)	840 (0.08)	905 (0.09)	896 (0.09)	977 (0.10)	1,169 (0.12)
Males	2,000 (0.19)	2,133 (0.20)	2,083 (0.20)	2,041 (0.19)	1,963 (0.19)	2,042 (0.19)	2,122 (0.20)
0 – 5 years	NA	NA	NA	NA	NA	NA	NA
6 – 12 years	NA	NA	NA	NA	NA	NA	NA
13 – 17 years	122 (0.03)	112 (0.03)	108 (0.03)	116 (0.03)	97 (0.02)	91 (0.02)	97 (0.02)
18 – 25 years	2,590 (0.43)	2,827 (0.47)	2,791 (0.45)	2,789 (0.45)	2,736 (0.44)	2,904 (0.46)	3,162 (0.50)

NA: not applicable

© 2024 Fond G et al. *JAMA Network Open*.

eTable 2. Relative-Risk (RR) Difference Between Annual Trends After vs Before the COVID-19 Pandemic, and Immediate Effect Estimation

	Immediate effect RR [95% CI]	p-value	Annual trends before the pandemic, RR [95% CI] (1)	p-value	Annual trends after the pandemic, RR [95% CI] (2)	p-value	RR diff (2) vs (1) [95% CI]	p-value
Outpatient psychiatric consultation	0.95 [0.86 – 1.05]	0.326	1.00 [0.99 – 1.02]	0.256	1.11 [1.06 – 1.17]	<0.001	1.10 [1.04 – 1.17]	<0.001
Females	0.99 [0.89 – 1.09]	0.817	1.02 [1.00 – 1.03]	<0.001	1.16 [1.10 – 1.22]	<0.001	1.13 [1.07 – 1.20]	<0.001
Males	0.92 [0.82 – 1.02]	0.099	0.99 [0.97 – 1.00]	0.342	1.05 [0.99 – 1.11]	0.061	1.06 [0.99 – 1.12]	0.067
0 – 5 years	0.88 [0.74 – 1.05]	0.156	0.93 [0.91 – 0.96]	<0.001	0.95 [0.87 – 1.04]	0.333	1.02 [0.91 – 1.13]	0.704
6 – 12 years	0.90 [0.78 – 1.04]	0.157	0.95 [0.93 – 0.97]	<0.001	1.00 [0.93 – 1.08]	0.873	1.05 [0.97 – 1.14]	0.200
13 – 17 years	0.97 [0.85 – 1.11]	0.642	0.99 [0.97 – 1.01]	0.442	1.14 [1.06 – 1.21]	<0.001	1.15 [1.06 – 1.23]	<0.001
18 – 25 years	0.98 [0.91 – 1.05]	0.538	1.05 [1.03 – 1.07]	<0.001	1.14 [1.10 – 1.19]	<0.001	1.08 [1.03 – 1.14]	<0.001
Psychiatric full-time hospitalization	0.88 [0.79 – 0.97]	0.013	1.01 [1.00 – 1.03]	0.016	1.10 [1.04 – 1.16]	<0.001	1.08 [1.02 – 1.15]	0.009
Females	0.93 [0.83 – 1.06]	0.277	1.04 [1.03 – 1.05]	<0.001	1.15 [1.07 – 1.22]	<0.001	1.09 [1.02 – 1.18]	0.010
Males	0.83 [0.76 – 0.90]	<0.001	0.99 [0.97 – 1.00]	0.260	1.04 [1.00 – 1.09]	0.033	1.05 [1.00 – 1.11]	0.040
0 – 5 years	0.57 [0.35 – 0.93]	0.025	0.89 [0.84 – 0.95]	<0.001	1.22 [0.98 – 1.52]	0.064	1.36 [1.08 – 1.73]	0.008
6 – 12 years	0.75 [0.58 – 0.98]	0.036	0.98 [0.95 – 1.01]	0.283	1.12 [1.00 – 1.27]	0.046	1.14 [1.00 – 1.31]	0.037
13 – 17 years	0.90 [0.77 – 1.04]	0.162	1.00 [0.99 – 1.02]	0.258	1.10 [1.01 – 1.19]	0.014	1.09 [1.00 – 1.19]	0.044
18 – 25 years	0.89 [0.83 – 0.95]	<0.001	1.01 [1.00 – 1.03]	0.007	1.09 [1.05 – 1.13]	<0.001	1.07 [1.02 – 1.12]	0.003
Hospitalization for suicide attempt	0.94 [0.81 – 1.11]	0.479	1.03 [1.01 – 1.05]	<0.001	1.15 [1.06 – 1.24]	<0.001	1.11 [1.01 – 1.21]	0.027
Females	0.96 [0.79 – 1.16]	0.676	1.03 [1.01 – 1.05]	0.002	1.18 [1.08 – 1.30]	<0.001	1.14 [1.02 – 1.27]	0.013
Males	0.91 [0.85 – 0.99]	0.022	1.03 [1.01 – 1.05]	<0.001	1.03 [0.99 – 1.08]	0.107	0.99 [0.94 – 1.05]	0.978
0 – 5 years	0.97 [0.80 – 1.17]	0.765	1.02 [0.96 – 1.09]	0.385	1.01 [0.93 – 1.09]	0.737	0.98 [0.89 – 1.08]	0.748

© 2024 Fond G et al. *JAMA Network Open*.

	Immediate effect RR [95% CI]	p-value	Annual trends before the pandemic, RR [95% CI] (1)	p-value	Annual trends after the pandemic, RR [95% CI] (2)	p-value	RR diff (2) vs (1) [95% CI]	p-value
6 – 12 years	1.00 [0.68 – 1.39]	0.988	1.08 [1.02 – 1.14]	0.003	1.12 [0.96 – 1.31]	0.124	1.04 [0.86 – 1.24]	0.665
13 – 17 years	0.99 [0.78 – 1.26]	0.955	1.01 [0.98 – 1.04]	0.274	1.15 [1.02 – 1.30]	0.016	1.13 [0.99 – 1.31]	0.068
18 – 25 years	0.89 [0.83 – 0.96]	0.002	1.04 [1.02 – 1.05]	<0.001	1.12 [1.08 – 1.16]	<0.001	1.07 [1.03 – 1.12]	<0.001
Antidepressants	1.00 [0.95 – 1.06]	0.943	1.09 [1.08 – 1.10]	<0.001	1.20 [1.17 – 1.23]	<0.001	1.10 [1.07 – 1.13]	<0.001
Females	1.02 [0.95 – 1.09]	0.616	1.10 [1.08 – 1.11]	<0.001	1.24 [1.21 – 1.28]	<0.001	1.13 [1.09 – 1.16]	<0.001
Males	0.98 [0.94 – 1.02]	0.348	1.08 [1.06 – 1.09]	<0.001	1.12 [1.10 – 1.14]	<0.001	1.03 [1.01 – 1.06]	<0.001
0 – 5 years	0.85 [0.81 – 0.90]	<0.001	0.95 [0.93 – 0.96]	<0.001	1.12 [1.09 – 1.15]	<0.001	1.17 [1.14 – 1.21]	<0.001
6 – 12 years	1.02 [0.92 – 1.13]	0.694	1.02 [1.01 – 1.04]	<0.001	1.14 [1.10 – 1.19]	<0.001	1.11 [1.07 – 1.15]	<0.001
13 – 17 years	1.03 [0.91 – 1.16]	0.664	1.09 [1.08 – 1.10]	<0.001	1.28 [1.21 – 1.35]	<0.001	1.17 [1.10 – 1.24]	<0.001
18 – 25 years	1.00 [0.95 – 1.04]	0.906	1.08 [1.06 – 1.10]	<0.001	1.17 [1.15 – 1.19]	<0.001	1.08 [1.05 – 1.10]	<0.001
Anxiolytics	1.02 [0.96 – 1.08]	0.562	1.02 [1.01 – 1.02]	<0.001	1.05 [1.02 – 1.08]	<0.001	1.03 [1.00 – 1.06]	0.033
Females	1.04 [0.96 – 1.12]	0.365	1.02 [1.01 – 1.03]	<0.001	1.07 [1.04 – 1.11]	<0.001	1.05 [1.01 – 1.09]	0.005
Males	0.99 [0.96 – 1.03]	0.755	1.01 [1.00 – 1.02]	<0.001	1.01 [0.99 – 1.03]	0.055	1.00 [0.97 – 1.02]	0.903
0 – 5 years	0.83 [0.76 – 0.92]	<0.001	0.93 [0.91 – 0.96]	<0.001	1.09 [1.03 – 1.16]	0.003	1.17 [1.08 – 1.25]	<0.001
6 – 12 years	1.01 [0.96 – 1.07]	0.653	0.98 [0.97 – 0.99]	<0.001	1.06 [1.03 – 1.09]	<0.001	1.08 [1.04 – 1.11]	<0.001
13 – 17 years	1.08 [0.95 – 1.24]	0.232	0.99 [0.98 – 1.00]	0.337	1.06 [0.99 – 1.12]	0.069	1.06 [0.99 – 1.14]	0.069
18 – 25 years	1.01 [0.96 – 1.06]	0.669	1.02 [1.01 – 1.04]	0.001	1.04 [1.02 – 1.06]	<0.001	1.01 [0.98 – 1.04]	0.438
Hypnotics	1.03 [1.00 – 1.07]	0.066	0.96 [0.95 – 0.98]	<0.001	1.04 [1.03 – 1.05]	<0.001	1.07 [1.05 – 1.09]	<0.001
Females	1.06 [1.02 – 1.09]	0.001	0.95 [0.94 – 0.97]	<0.001	1.06 [1.05 – 1.07]	<0.001	1.11 [1.09 – 1.13]	<0.001

© 2024 Fond G et al. *JAMA Network Open*.

	Immediate effect RR [95% CI]	p-value	Annual trends before the pandemic, RR [95% CI] (1)	p-value	Annual trends after the pandemic, RR [95% CI] (2)	p-value	RR diff (2) vs (1) [95% CI]	p-value
Males	1.00 [0.96 – 1.05]	0.821	0.98 [0.96 – 0.99]	0.029	1.01 [0.99 – 1.02]	0.106	1.03 [1.00 – 1.05]	0.007
0 – 5 years	0.77 [0.68 – 0.88]	<0.001	1.10 [1.06 – 1.13]	<0.001	1.17 [1.09 – 1.25]	<0.001	1.06 [0.99 – 1.14]	0.072
6 – 12 years	0.94 [0.80 – 1.10]	0.420	1.09 [1.06 – 1.12]	<0.001	1.04 [0.98 – 1.11]	0.139	0.96 [0.89 – 1.03]	0.267
13 – 17 years	1.06 [0.97 – 1.16]	0.222	0.95 [0.93 – 0.97]	<0.001	1.04 [1.00 – 1.09]	0.036	1.09 [1.04 – 1.15]	<0.001
18 – 25 years	1.06 [1.03 – 1.10]	<0.001	0.94 [0.92 – 0.96]	<0.001	1.02 [1.01 – 1.03]	<0.001	1.08 [1.06 – 1.10]	<0.001
Mood stabilizers	0.98 [0.96 – 1.01]	0.184	1.00 [1.00 – 1.01]	0.002	1.03 [1.03 – 1.04]	<0.001	1.02 [1.02 – 1.03]	<0.001
Females	0.99 [0.96 – 1.02]	0.353	1.00 [1.00 – 1.01]	0.007	1.06 [1.06 – 1.07]	<0.001	1.05 [1.04 – 1.06]	<0.001
Males	0.98 [0.96 – 1.00]	0.100	1.00 [0.99 – 1.00]	0.052	1.01 [1.00 – 1.01]	0.002	1.00 [0.99 – 1.01]	0.147
0 – 5 years	0.96 [0.92 – 1.00]	0.052	0.94 [0.93 – 0.95]	<0.001	1.01 [0.99 – 1.03]	0.140	1.07 [1.04 – 1.10]	<0.001
6 – 12 years	0.99 [0.96 – 1.02]	0.464	0.98 [0.97 – 0.98]	<0.001	0.99 [0.98 – 1.00]	0.094	1.01 [1.00 – 1.02]	0.008
13 – 17 years	0.99 [0.97 – 1.02]	0.497	0.98 [0.98 – 0.99]	<0.001	1.01 [1.01 – 1.02]	<0.001	1.03 [1.02 – 1.03]	<0.001
18 – 25 years	0.98 [0.96 – 1.01]	0.190	1.03 [1.02 – 1.04]	<0.001	1.05 [1.04 – 1.06]	<0.001	1.01 [1.00 – 1.03]	0.009
Antipsychotics	0.98 [0.95 – 1.01]	0.148	1.06 [1.05 – 1.07]	<0.001	1.10 [1.09 – 1.11]	<0.001	1.03 [1.02 – 1.05]	<0.001
Females	1.00 [0.95 – 1.04]	0.872	1.09 [1.08 – 1.09]	<0.001	1.19 [1.17 – 1.21]	<0.001	1.09 [1.07 – 1.11]	<0.001
Males	0.98 [0.96 – 1.00]	0.046	1.04 [1.04 – 1.05]	<0.001	1.04 [1.03 – 1.05]	<0.001	0.99 [0.98 – 1.00]	0.916
0 – 5 years	1.01 [0.93 – 1.10]	0.746	1.02 [1.00 – 1.03]	0.008	1.10 [1.06 – 1.13]	<0.001	1.07 [1.04 – 1.11]	<0.001
6 – 12 years	0.97 [0.93 – 1.02]	0.279	1.05 [1.04 – 1.06]	<0.001	1.10 [1.08 – 1.12]	<0.001	1.04 [1.02 – 1.06]	<0.001
13 – 17 years	0.99 [0.94 – 1.05]	0.839	1.04 [1.03 – 1.05]	<0.001	1.12 [1.09 – 1.14]	<0.001	1.07 [1.04 – 1.09]	<0.001
18 – 25 years	0.97 [0.95 – 1.00]	0.050	1.06 [1.04 – 1.08]	<0.001	1.08 [1.08 – 1.08]	<0.001	1.02 [1.00 – 1.03]	0.031

© 2024 Fond G et al. *JAMA Network Open*.

	Immediate effect RR [95% CI]	p-value	Annual trends before the pandemic, RR [95% CI] (1)	p-value	Annual trends after the pandemic, RR [95% CI] (2)	p-value	RR diff (2) vs (1) [95% CI]	p-value
Methylphenidate	0.89 [0.85 – 0.93]	**<0.001**	1.08 [1.07 – 1.09]	**<0.001**	1.19 [1.17 – 1.22]	**<0.001**	1.10 [1.08 – 1.13]	**<0.001**
Females	0.87 [0.83 – 0.90]	**<0.001**	1.09 [1.08 – 1.10]	**<0.001**	1.26 [1.24 – 1.29]	**<0.001**	1.15 [1.13 – 1.18]	**<0.001**
Males	0.89 [0.85 – 0.93]	**<0.001**	1.07 [1.06 – 1.08]	**<0.001**	1.17 [1.15 – 1.20]	**<0.001**	1.09 [1.06 – 1.12]	**<0.001**
0 – 5 years	0.93 [0.84 – 1.02]	0.101	1.05 [1.03 – 1.07]	**<0.001**	1.13 [1.08 – 1.17]	**<0.001**	1.07 [1.02 – 1.12]	**0.001**
6 – 12 years	0.91 [0.87 – 0.95]	**<0.001**	1.07 [1.06 – 1.08]	**<0.001**	1.18 [1.15 – 1.20]	**<0.001**	1.09 [1.07 – 1.12]	**<0.001**
13 – 17 years	0.86 [0.81 – 0.90]	**<0.001**	1.08 [1.06 – 1.09]	**<0.001**	1.18 [1.15 – 1.21]	**<0.001**	1.09 [1.07 – 1.12]	**<0.001**
18 – 25 years	0.84 [0.80 – 0.88]	**<0.001**	1.12 [1.10 – 1.13]	**<0.001**	1.33 [1.31 – 1.36]	**<0.001**	1.19 [1.16 – 1.22]	**<0.001**
Drugs used in alcohol dependence	0.89 [0.84 – 0.95]	**<0.001**	1.02 [1.00 – 1.05]	**0.047**	1.13 [1.10 – 1.16]	**<0.001**	1.10 [1.06 – 1.14]	**<0.001**
Females	0.86 [0.80 – 0.93]	**<0.001**	1.06 [1.03 – 1.09]	**<0.001**	1.19 [1.17 – 1.21]	**<0.001**	1.12 [1.08 – 1.16]	**<0.001**
Males	0.91 [0.86 – 0.97]	**0.004**	1.01 [0.98 – 1.04]	0.467	1.09 [1.06 – 1.13]	**<0.001**	1.08 [1.04 – 1.13]	**<0.001**
0 – 5 years	NA	NA	NA	NA	NA	NA	NA	NA
6 – 12 years	NA	NA	NA	NA	NA	NA	NA	NA
13 – 17 years	0.72 [0.59 – 0.87]	**<0.001**	1.05 [0.99 – 1.12]	0.099	1.08 [0.97 – 1.19]	0.133	1.02 [0.90 – 1.15]	0.686
18 – 25 years	0.90 [0.84 – 0.96]	**0.002**	1.01 [0.98 – 1.05]	0.238	1.12 [1.09 – 1.15]	**<0.001**	1.10 [1.06 – 1.15]	**<0.001**

In bold: statistical significance. NA: not applicable

© 2024 Fond G et al. *JAMA Network Open*.

eTable 3. Change in Prescription Rates Between 2016 and 2022 by Drug

	All		Males		Females		0-5 years		6-12 years		13-17 years		18-25 years	
	2016	2022	2016	2022	2016	2022	2016	2022	2016	2022	2016	2022	2016	2022
Whole population	20,829,566	20,697,169	10,621,339	10,593,993	10,208,227	10,103,176	4,695,667	4,277,675	5,864,399	5,769,970	4,177,541	4,309,077	6,091,959	6,340,447
**Mood stabilizers**														
N03AF01 (Carbamazepine), N	10,145	10,340	5,754	5,627	4,391	4,713	1,030	1,132	2,322	2,255	2,624	2,477	4,843	5,172
Rates (/1000)	0.49	0.50	0.54	0.53	0.43	0.47	0.22	0.26	0.40	0.39	0.63	0.57	0.79	0.82
Change in rates	3%		-2%		8%		21%		-1%		-8%		3%	
N03AG01 (Valproic acid), N	43,714	36,132	27,680	25,549	16,037	10,583	8,625	5,642	12,153	9,380	9,582	7,830	16,566	15,908
Rates (/1000)	2.10	1.75	2.61	2.41	1.57	1.05	1.84	1.32	2.07	1.63	2.29	1.82	2.72	2.51
Change in rates	-17%		-7%		-33%		-28%		-22%		-21%		-8%	
N03AX09 (Lamotrigine), N	26,631	39,198	10,143	13,097	16,488	26,101	1,344	1,218	4,832	5,041	7,052	8,745	15,222	26,499
Rates (/1000)	1.28	1.89	0.95	1.24	1.62	2.58	0.29	0.28	0.82	0.87	1.69	2.03	2.50	4.18
Change in rates	48%		29%		60%		-1%		6%		20%		67%	
N05AN01 (Lithium), N	2,525	6,391	1,128	2,131	1,397	4,260	22	19	43	79	294	719	2,219	5,755
Rates (/1000)	0.12	0.31	0.11	0.20	0.14	0.42	0.00	0.00	0.01	0.01	0.07	0.17	0.36	0.91
Change in rates	155%		89%		208%		-5%		87%		137%		149%	
**Antipsychotics**														
N05AA01 (Chlorpromazine), N	1,850	4,024	822	1,484	1,028	2,540	33	35	149	279	364	1,129	1,350	2,661
Rates (/1000)	0.09	0.19	0.08	0.14	0.10	0.25	0.01	0.01	0.03	0.05	0.09	0.26	0.22	0.42
Change in rates	119%		81%		150%		16%		90%		201%		89%	
N05AA02 (Levomepronazine), N	2,951	4,466	1,937	2,412	1,014	2,054	58	32	433	593	825	1,576	1,750	2,451
Rates (/1000)	0.14	0.22	0.18	0.23	0.10	0.20	0.01	0.01	0.07	0.10	0.20	0.37	0.29	0.39
Change in rates	52%		25%		105%		-39%		39%		85%		35%	
N05AA06 (Cyamemazine), N	37,779	66,331	23,852	31,191	13,928	35,140	615	742	6,223	8,899	13,941	26,170	18,546	33,131
Rates (/1000)	1.81	3.20	2.25	2.94	1.36	3.48	0.13	0.17	1.06	1.54	3.34	6.07	3.04	5.23
Change in rates	77%		31%		155%		32%		45%		82%		72%	
N05AC01 (Periciazine), N	1,891	1,512	1,376	1,072	515	440	142	82	649	555	553	455	655	521
Rates (/1000)	0.09	0.07	0.13	0.10	0.05	0.04	0.03	0.02	0.11	0.10	0.13	0.11	0.11	0.08
Change in rates	-20%		-22%		-14%		-37%		-13%		-20%		-24%	
N05AD01 (Haloperidol), N	4,511	4,464	3,108	3,016	1,403	1,448	57	29	488	414	903	926	3,205	3,244
Rates (/1000)	0.22	0.22	0.29	0.28	0.14	0.14	0.01	0.01	0.08	0.07	0.22	0.21	0.53	0.51

© 2024 Fond G et al. *JAMA Network Open*.

	All		Males		Females		0-5 years		6-12 years		13-17 years		18-25 years	
	2016	2022	2016	2022	2016	2022	2016	2022	2016	2022	2016	2022	2016	2022
Whole population	20,829,566	20,697,169	10,621,339	10,593,993	10,208,227	10,103,176	4,695,667	4,277,675	5,864,399	5,769,970	4,177,541	4,309,077	6,091,959	6,340,447
Change in rates	0%		-3%		4%		-44%		-14%		-1%		-3%	
N05AD05 (Pipamperone), N	1,823	1,234	1,287	867	536	367	53	36	412	255	554	304	892	702
Rates (/1000)	0.09	0.06	0.12	0.08	0.05	0.04	0.01	0.01	0.07	0.04	0.13	0.07	0.15	0.11
Change in rates	-32%		-32%		-31%		-25%		-37%		-47%		-24%	
N05AF05 (Zuclopenthixol), N	1,949	2,124	1,521	1,537	428	587	1	2	93	65	547	451	1,397	1,692
Rates (/1000)	0.09	0.10	0.14	0.15	0.04	0.06	0.00	0.00	0.02	0.01	0.13	0.10	0.23	0.27
Change in rates	10%		1%		39%		120%		-29%		-20%		16%	
N05AH01 (Loxapine), N	8,887	15,859	5,935	8,570	2,952	7,290	7	12	238	445	1,811	3,306	7,017	12,476
Rates (/1000)	0.43	0.77	0.56	0.81	0.29	0.72	0.00	0.00	0.04	0.08	0.43	0.77	1.15	1.97
Change in rates	80%		45%		150%		88%		90%		77%		71%	
N05AH02 (Clozapine), N	1,401	2,767	965	1,742	436	1,025		4	7	22	123	326	1,300	2,488
Rates (/1000)	0.07	0.13	0.09	0.16	0.04	0.10		0.00	0.00	0.00	0.03	0.08	0.21	0.39
Change in rates	99%		81%		138%		NA		219%		157%		84%	
N05AH03 (Olanzapine), N	8,908	13,010	5,611	6,669	3,297	6,341	43	32	134	181	1,347	2,236	7,577	10,871
Rates (/1000)	0.43	0.63	0.53	0.63	0.32	0.63	0.01	0.01	0.02	0.03	0.32	0.52	1.24	1.71
Change in rates	47%		19%		94%		-18%		37%		61%		38%	
N05AH04 (Quetiapine), N	8,499	20,412	4,132	6,703	4,368	13,711	31	48	72	140	959	3,294	7,602	17,438
Rates (/1000)	0.41	0.99	0.39	0.63	0.43	1.36	0.01	0.01	0.01	0.02	0.23	0.76	1.25	2.75
Change in rates	142%		63%		217%		70%		98%		233%		120%	
N05AL01 (Sulpiride), N	2,565	1,608	1,054	617	1,511	991	56	27	241	103	535	288	1,754	1,203
Rates (/1000)	0.12	0.08	0.10	0.06	0.15	0.10	0.01	0.01	0.04	0.02	0.13	0.07	0.29	0.19
Change in rates	-37%		-41%		-34%		-47%		-57%		-48%		-34%	
N05AL03 (Tiapride), N	2,759	1,881	1,886	1,178	873	703	63	16	476	287	719	395	1,573	1,235
Rates (/1000)	0.13	0.09	0.18	0.11	0.09	0.07	0.01	0.00	0.08	0.05	0.17	0.09	0.26	0.19
Change in rates	-31%		-37%		-19%		-72%		-39%		-47%		-25%	
N05AL05 (Amisulpride), N	3,159	3,878	2,007	2,047	1,152	1,831	13	8	20	28	389	417	2,779	3,474
Rates (/1000)	0.15	0.19	0.19	0.19	0.11	0.18	0.00	0.00	0.00	0.00	0.09	0.10	0.46	0.55
Change in rates	24%		2%		61%		-32%		42%		4%		20%	
N05AX08 (Risperidone), N	52,014	66,171	38,976	46,359	13,041	19,813	1,445	1,927	15,618	21,242	18,561	22,288	19,766	24,899
Rates (/1000)	2.50	3.20	3.67	4.38	1.28	1.96	0.31	0.45	2.66	3.68	4.44	5.17	3.24	3.93

© 2024 Fond G et al. *JAMA Network Open*.

	All		Males		Females		0-5 years		6-12 years		13-17 years		18-25 years	
	2016	2022	2016	2022	2016	2022	2016	2022	2016	2022	2016	2022	2016	2022
Whole population	20,829,566	20,697,169	10,621,339	10,593,993	10,208,227	10,103,176	4,695,667	4,277,675	5,864,399	5,769,970	4,177,541	4,309,077	6,091,959	6,340,447
Change in rates	28%		19%		54%		46%		38%		16%		21%	
N05AX12 (Aripiprazole), N	21,480	45,643	12,770	21,744	8,710	23,899	55	145	1,618	4,491	6,349	14,311	14,460	28,712
Rates (/1000)	1.03	2.21	1.20	2.05	0.85	2.37	0.01	0.03	0.28	0.78	1.52	3.32	2.37	4.53
Change in rates	114%		71%		177%		189%		182%		119%		91%	
N05AX13 (Paliperidone), N	3,861	5,103	3,188	4,249	673	854	4	4	4	7	341	352	3,600	4,833
Rates (/1000)	0.19	0.25	0.30	0.40	0.07	0.08	0.00	0.00	0.00	0.00	0.08	0.08	0.59	0.76
Change in rates	33%		34%		28%		10%		78%		0%		29%	
**Anxiolytics**														
N05BA01 (Diazepam), N	57,632	74,825	28,168	34,747	29,465	40,078	13,983	8,015	5,127	3,764	6,816	8,827	32,023	54,732
Rates (/1000)	2.77	3.62	2.65	3.28	2.89	3.97	2.98	1.87	0.87	0.65	1.63	2.05	5.26	8.63
Change in rates	31%		24%		37%		-37%		-25%		26%		64%	
N05BA04 (Oxazepam), N	32,159	74,937	13,037	24,882	19,122	50,055	182	254	274	439	3,748	8,505	28,134	66,213
Rates (/1000)	1.54	3.62	1.23	2.35	1.87	4.95	0.04	0.06	0.05	0.08	0.90	1.97	4.62	10.44
Change in rates	135%		91%		164%		53%		63%		120%		126%	
N05BA05 (Potassium clorazepate), N	4,633	4,347	2,184	1,769	2,449	2,578	25	14	170	109	757	615	3,727	3,653
Rates (/1000)	0.22	0.21	0.21	0.17	0.24	0.26	0.01	0.00	0.03	0.02	0.18	0.14	0.61	0.58
Change in rates	-6%		-19%		6%		-39%		-35%		-21%		-6%	
N05BA06 (Lorazepam), N	10,277	18,860	4,259	6,691	6,018	12,169	58	50	124	173	1,406	2,704	8,774	16,152
Rates (/1000)	0.49	0.91	0.40	0.63	0.59	1.20	0.01	0.01	0.02	0.03	0.34	0.63	1.44	2.55
Change in rates	85%		58%		104%		-5%		42%		86%		77%	
N05BA08 (Bromazepam), N	46,872	42,435	15,625	13,371	31,248	29,064	255	150	704	510	6,661	5,382	39,409	36,591
Rates (/1000)	2.25	2.05	1.47	1.26	3.06	2.88	0.05	0.04	0.12	0.09	1.59	1.25	6.47	5.77
Change in rates	-9%		-14%		-6%		-35%		-26%		-22%		-11%	
N05BA09 (Clobazam), N	18,055	17,506	8,846	8,629	9,209	8,877	1,085	788	3,189	2,811	4,158	3,958	10,334	10,603
Rates (/1000)	0.87	0.85	0.83	0.81	0.90	0.88	0.23	0.18	0.54	0.49	1.00	0.92	1.70	1.67
Change in rates	-2%		-2%		-3%		-20%		-10%		-8%		-1%	
N05BA11 (Prazepam), N	36,491	41,216	12,076	12,094	24,415	29,123	194	130	1,051	729	6,822	7,408	28,692	33,331
Rates (/1000)	1.75	1.99	1.14	1.14	2.39	2.88	0.04	0.03	0.18	0.13	1.63	1.72	4.71	5.26
Change in rates	14%		0%		21%		-26%		-30%		5%		12%	

© 2024 Fond G et al. *JAMA Network Open*.

	All		Males		Females		0-5 years		6-12 years		13-17 years		18-25 years	
	2016	2022	2016	2022	2016	2022	2016	2022	2016	2022	2016	2022	2016	2022
Whole population	20,829,566	20,697,169	10,621,339	10,593,993	10,208,227	10,103,176	4,695,667	4,277,675	5,864,399	5,769,970	4,177,541	4,309,077	6,091,959	6,340,447
N05BA12 (Alprazolam), N	153,582	221,022	51,440	66,199	102,142	154,824	632	556	1,517	1,600	22,722	31,173	129,599	189,468
Rates (/1000)	7.37	10.68	4.84	6.25	10.01	15.32	0.13	0.13	0.26	0.28	5.44	7.23	21.27	29.88
Change in rates	45%		29%		53%		-3%		7%		33%		40%	
N05BA21 (Clotiazepam), N	11,918	13,512	3,841	3,914	8,077	9,598	40	37	100	85	1,767	1,960	10,061	11,496
Rates (/1000)	0.57	0.65	0.36	0.37	0.79	0.95	0.01	0.01	0.02	0.01	0.42	0.45	1.65	1.81
Change in rates	14%		2%		20%		2%		-14%		8%		10%	
N05BB01 (Hydroxyzine), N	256,684	293,117	109,819	112,298	146,865	180,819	40,848	33,690	50,868	56,689	62,719	80,199	104,116	125,343
Rates (/1000)	12.32	14.16	10.34	10.60	14.39	17.90	8.70	7.88	8.67	9.82	15.01	18.61	17.09	19.77
Change in rates	15%		3%		24%		-9%		13%		24%		16%	
N05BE01 (Buspirone), N	4,017	5,454	1,393	1,672	2,624	3,782	10	10	40	66	724	1,075	3,272	4,359
Rates (/1000)	0.19	0.26	0.13	0.16	0.26	0.37	0.00	0.00	0.01	0.01	0.17	0.25	0.54	0.69
Change in rates	37%		20%		46%		10%		68%		44%		28%	
**Hypnotics excluding melatonin**														
N05CD06 (Lormetazepam), N	10,311	15,919	4,332	5,781	5,979	10,138	52	49	91	128	909	1,165	9,321	14,673
Rates (/1000)	0.50	0.77	0.41	0.55	0.59	1.00	0.01	0.01	0.02	0.02	0.22	0.27	1.53	2.31
Change in rates	55%		34%		71%		3%		43%		24%		51%	
N05CD08 (Midazolam), N	19,658	35,073	10,751	19,497	8,907	15,576	7,362	12,698	7,773	13,079	4,099	7,555	792	2,277
Rates (/1000)	0.94	1.69	1.01	1.84	0.87	1.54	1.57	2.97	1.33	2.27	0.98	1.75	0.13	0.36
Change in rates	80%		82%		77%		89%		71%		79%		176%	
N05CD11 (Loprazolam), N	3,369	3,262	1,313	1,176	2,056	2,086	13	15	44	25	340	230	2,988	2,999
Rates (/1000)	0.16	0.16	0.12	0.11	0.20	0.21	0.00	0.00	0.01	0.00	0.08	0.05	0.49	0.47
Change in rates	-3%		-10%		3%		27%		-42%		-34%		-4%	
N05CF01 (Zoplicone), N	35,889	57,031	14,791	21,471	21,098	35,560	158	199	232	293	3,848	4,653	31,824	52,167
Rates (/1000)	1.72	2.76	1.39	2.03	2.07	3.52	0.03	0.05	0.04	0.05	0.92	1.08	5.22	8.23
Change in rates	60%		46%		70%		38%		28%		17%		57%	
N05CF02 (Zolpidem), N	56,484	9,465	21,348	3,616	35,136	5,849	291	59	421	102	5,160	784	50,846	8,562
Rates (/1000)	2.71	0.46	2.01	0.34	3.44	0.58	0.06	0.01	0.07	0.02	1.24	0.18	8.35	1.35
Change in rates	-83%		-83%		-83%		-78%		-75%		-85%		-84%	

© 2024 Fond G et al. *JAMA Network Open*.

	All		Males		Females		0-5 years		6-12 years		13-17 years		18-25 years	
	2016	2022	2016	2022	2016	2022	2016	2022	2016	2022	2016	2022	2016	2022
Whole population	20,829,566	20,697,169	10,621,339	10,593,993	10,208,227	10,103,176	4,695,667	4,277,675	5,864,399	5,769,970	4,177,541	4,309,077	6,091,959	6,340,447
**Antidepressants**														
N06AA04 (Clomipramine), N	5,269	4,937	2,804	2,046	2,465	2,891	99	36	1,412	617	1,144	965	2,727	3,425
Rates (/1000)	0.25	0.24	0.26	0.19	0.24	0.29	0.02	0.01	0.24	0.11	0.27	0.22	0.45	0.54
Change in rates	-6%		-27%		19%		-60%		-56%		-18%		21%	
N06AA09 (Amitriptyline), N	28,767	35,760	9,836	10,581	18,931	25,180	777	512	3,895	3,373	7,463	8,068	17,115	24,393
Rates (/1000)	1.38	1.73	0.93	1.00	1.85	2.49	0.17	0.12	0.66	0.58	1.79	1.87	2.81	3.85
Change in rates	25%		8%		34%		-28%		-12%		5%		37%	
N06AB03 (Fluoxetine), N	23,585	61,383	7,609	16,015	15,976	45,368	123	167	945	2,264	6,348	21,589	16,813	39,567
Rates (/1000)	1.13	2.97	0.72	1.51	1.57	4.49	0.03	0.04	0.16	0.39	1.52	5.01	2.76	6.24
Change in rates	162%		111%		187%		49%		143%		230%		126%	
N06AB04 (Citalopram), N	6,481	4,123	2,245	1,213	4,236	2,910	81	24	96	52	783	508	5,612	3,587
Rates (/1000)	0.31	0.20	0.21	0.11	0.41	0.29	0.02	0.01	0.02	0.01	0.19	0.12	0.92	0.57
Change in rates	-36%		-46%		-31%		-67%		-45%		-37%		-39%	
N06AB05 (Paroxetine), N	37,749	72,791	14,398	22,970	23,351	49,822	298	419	420	576	4,553	8,612	33,021	64,370
Rates (/1000)	1.81	3.52	1.36	2.17	2.29	4.93	0.06	0.10	0.07	0.10	1.09	2.00	5.42	10.15
Change in rates	94%		60%		116%		54%		39%		83%		87%	
N06AB06 (Sertraline), N	32,075	100,131	12,767	29,623	19,308	70,510	156	285	1,947	4,229	12,098	36,524	19,301	63,514
Rates (/1000)	1.54	4.84	1.20	2.80	1.89	6.98	0.03	0.07	0.33	0.73	2.90	8.48	3.17	10.02
Change in rates	214%		133%		269%		101%		121%		193%		216%	
N06AB10 (Escitalopram), N	55,902	65,604	18,516	18,707	37,387	46,897	522	371	718	638	6,515	7,536	48,908	57,997
Rates (/1000)	2.68	3.17	1.74	1.77	3.66	4.64	0.11	0.09	0.12	0.11	1.56	1.75	8.03	9.15
Change in rates	18%		1%		27%		-22%		-10%		12%		14%	
N06AX03 (Mianserine), N	8,157	12,724	3,169	4,220	4,988	8,504	44	45	120	116	1,223	1,734	6,861	10,997
Rates (/1000)	0.39	0.61	0.30	0.40	0.49	0.84	0.01	0.01	0.02	0.02	0.29	0.40	1.13	1.73
Change in rates	57%		34%		72%		12%		-2%		37%		54%	
N06AX11 (Mirtazapine), N	7,168	15,872	3,165	5,891	4,003	9,981	37	45	61	91	824	1,817	6,345	14,140
Rates (/1000)	0.34	0.77	0.30	0.56	0.39	0.99	0.01	0.01	0.01	0.02	0.20	0.42	1.04	2.23
Change in rates	123%		87%		152%		34%		52%		114%		114%	

© 2024 Fond G et al. *JAMA Network Open*.

	All		Males		Females		0-5 years		6-12 years		13-17 years		18-25 years	
	2016	2022	2016	2022	2016	2022	2016	2022	2016	2022	2016	2022	2016	2022
Whole population	20,829,566	20,697,169	10,621,339	10,593,993	10,208,227	10,103,176	4,695,667	4,277,675	5,864,399	5,769,970	4,177,541	4,309,077	6,091,959	6,340,447
N06AX16 (Venlafaxine), N	20,939	42,060	7,606	12,918	13,333	29,142	231	215	299	347	2,031	4,261	18,648	37,952
Rates (/1000)	1.01	2.03	0.72	1.22	1.31	2.88	0.05	0.05	0.05	0.06	0.49	0.99	3.06	5.99
Change in rates	102%		70%		121%		2%		18%		103%		96%	
N06AX21 (Duloxetine), N	5,898	8,186	2,088	2,361	3,810	5,825	61	61	110	103	534	692	5,249	7,408
Rates (/1000)	0.28	0.40	0.20	0.22	0.37	0.58	0.01	0.01	0.02	0.02	0.13	0.16	0.86	1.17
Change in rates	40%		13%		54%		10%		-5%		26%		36%	
N06AX22 (Agomelatine), N	1,996	362	738	138	1,258	224	12		31	3	157	54	1,814	310
Rates (/1000)	0.10	0.02	0.07	0.01	0.12	0.02	0.00		0.01	0.00	0.04	0.01	0.30	0.05
Change in rates	-82%		-81%		-82%		-100%		-90%		-67%		-84%	
**Alcohol dependence drugs**														
N07BB03 (Acamprosate), N	909	1,281	692	919	217	362	9	2	6	10	31	27	863	1,242
Rates (/1000)	0.04	0.06	0.07	0.09	0.02	0.04	0.00	0.00	0.00	0.00	0.01	0.01	0.14	0.20
Change in rates	42%		33%		69%		-76%		69%		-16%		38%	
N07BB04 (Naltrexone), N	691	983	445	530	246	453	4	3	11	9	55	43	629	935
Rates (/1000)	0.03	0.05	0.04	0.05	0.02	0.04	0.00	0.00	0.00	0.00	0.01	0.01	0.10	0.15
Change in rates	43%		19%		86%		-18%		-17%		-24%		43%	
N07BB05 (Nalmefene), N	1,289	1,002	951	640	338	362	7	3	14	6	36	21	1,233	973
Rates (/1000)	0.06	0.05	0.09	0.06	0.03	0.04	0.00	0.00	0.00	0.00	0.01	0.00	0.20	0.15
Change in rates	-22%		-33%		8%		-53%		-56%		-43%		-24%	

NA: not applicable (division by zero).

© 2024 Fond G et al. *JAMA Network Open*.

eFigure 1. Sex-Specific Trends in Mental Health Care and Prescriptions Before and After the Beginning of the COVID-19 Pandemic With Annual Relative Risk

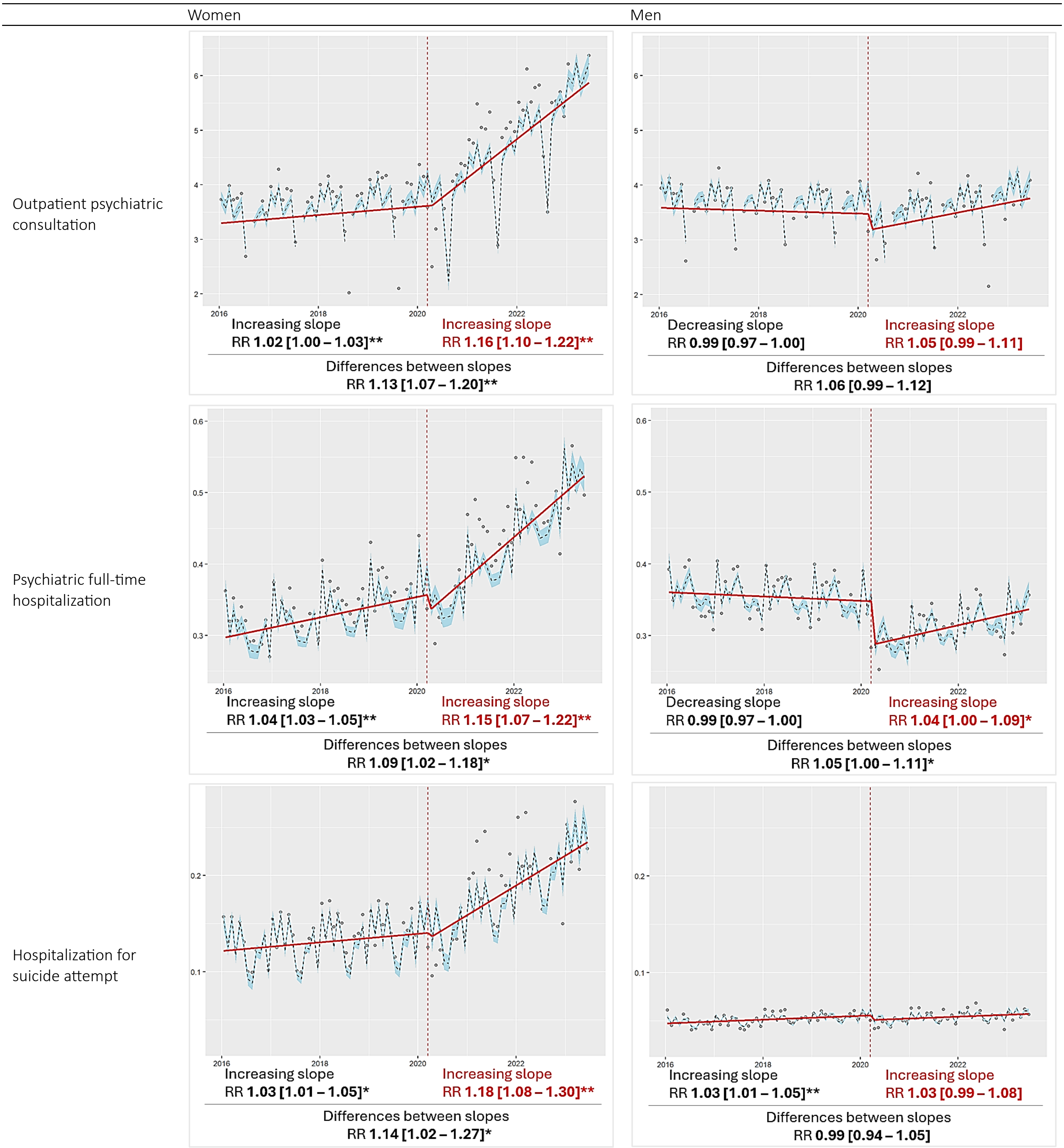

© 2024 Fond G et al. *JAMA Network Open*.

Women | Men

Antidepressants

Women

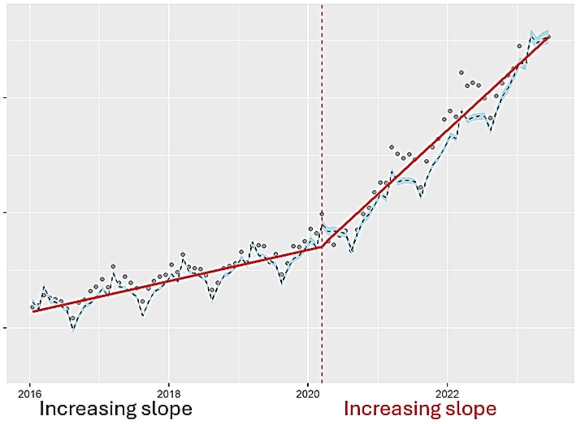

Increasing slope RR **1.10 [1.08 – 1.11]****

Increasing slope RR **1.24 [1.21 – 1.28]****

Differences between slopes RR **1.13 [1.09 – 1.16]****

Men

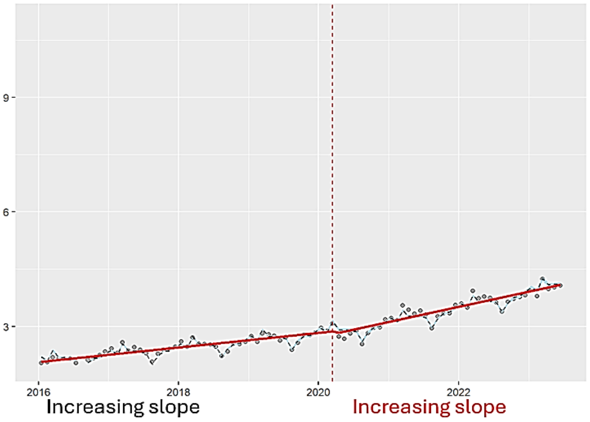

Increasing slope RR **1.08 [1.06 – 1.09]****

Increasing slope RR **1.12 [1.10 – 1.14]****

Differences between slopes RR **1.03 [1.01 – 1.06]****

Anxiolytics

Women

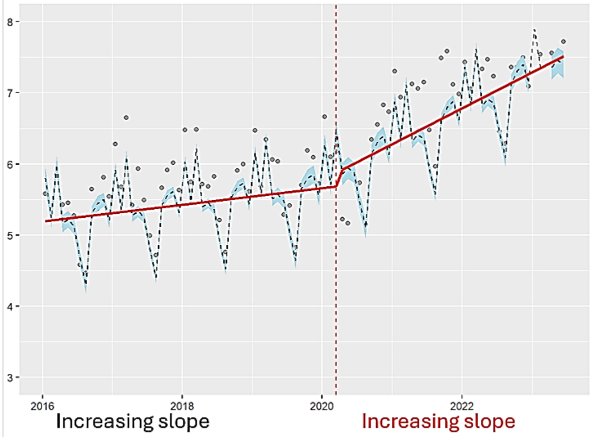

Increasing slope RR **1.02 [1.01 – 1.03]****

Increasing slope RR **1.07 [1.04 – 1.11]****

Differences between slopes RR **1.05 [1.01 – 1.09]***

Men

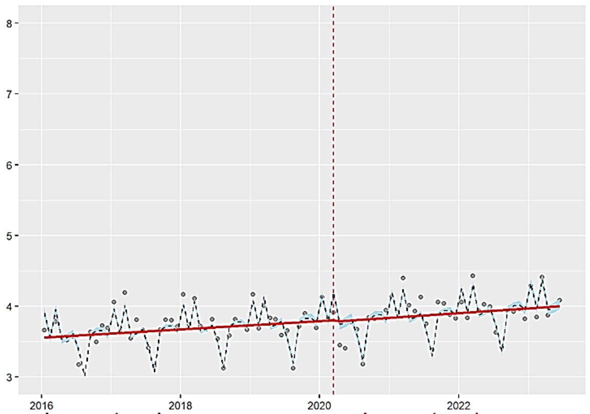

Increasing slope RR **1.01 [1.00 – 1.02]****

Increasing slope RR **1.01 [0.99 – 1.03]**

Differences between slopes RR **1.00 [0.97 – 1.02]**

Hypnotics

Women

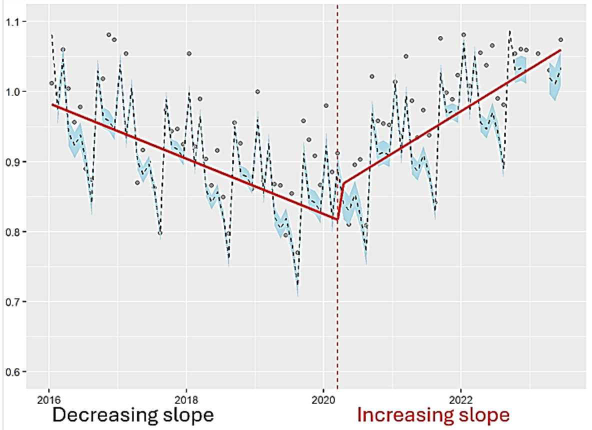

Decreasing slope RR **0.95 [0.94 – 0.97]****

Increasing slope RR **1.06 [1.05 – 1.07]****

Differences between slopes RR **1.11 [1.09 – 1.13]****

Men

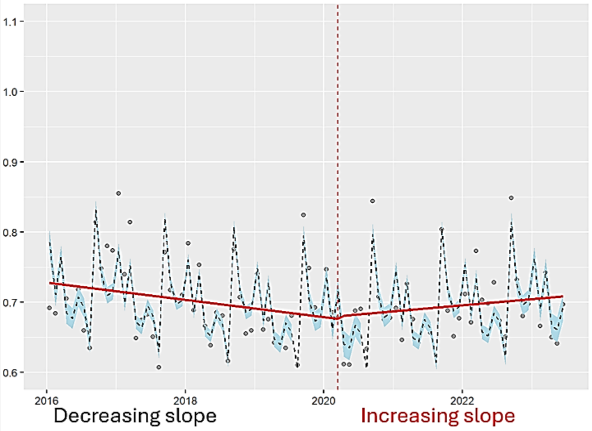

Decreasing slope RR **0.98 [0.96 – 0.99]***

Increasing slope RR **1.01 [0.99 – 1.02]**

Differences between slopes RR **1.03 [1.00 – 1.05]***

© 2024 Fond G et al. *JAMA Network Open*.

	Women	Men

Mood stabilizers

Women

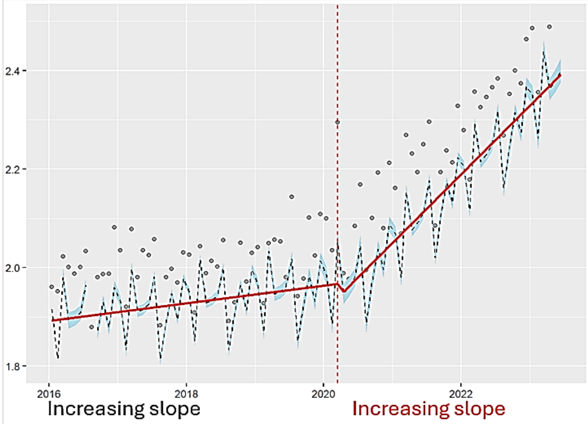

Increasing slope
RR **1.00 [1.00 – 1.01]***

Increasing slope
RR **1.06 [1.06 – 1.07]****

Differences between slopes
RR **1.05 [1.04 – 1.06]****

Men

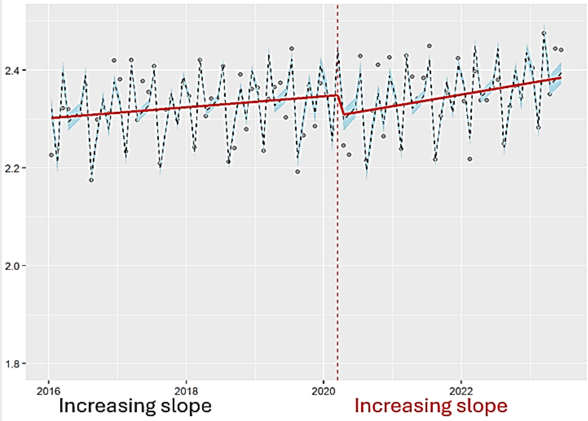

Increasing slope
RR **1.00 [0.99 – 1.00]**

Increasing slope
RR **1.01 [1.00 – 1.01]***

Differences between slopes
RR **1.00 [0.99 – 1.01]**

Antipsychotics

Women

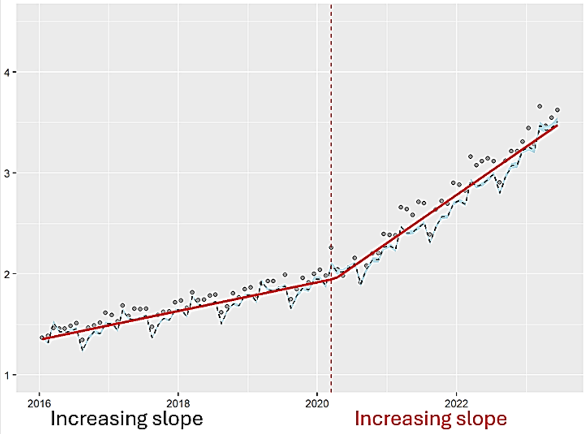

Increasing slope
RR **1.09 [1.08 – 1.09]****

Increasing slope
RR **1.19 [1.17 – 1.21]****

Differences between slopes
RR **1.09 [1.07 – 1.11]****

Men

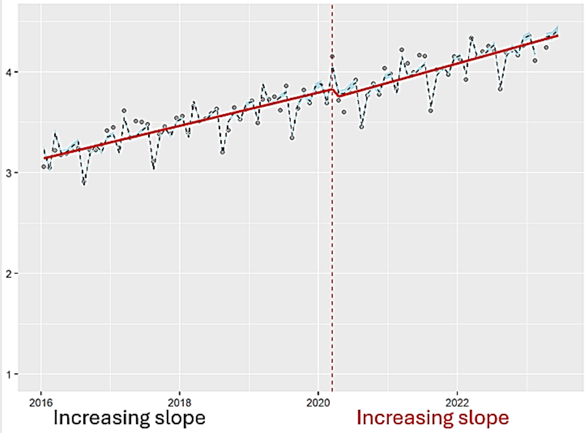

Increasing slope
RR **1.04 [1.04 – 1.05]****

Increasing slope
RR **1.04 [1.03 – 1.05]****

Differences between slopes
RR **0.99 [0.98 – 1.00]**

Methylphenidate

Women

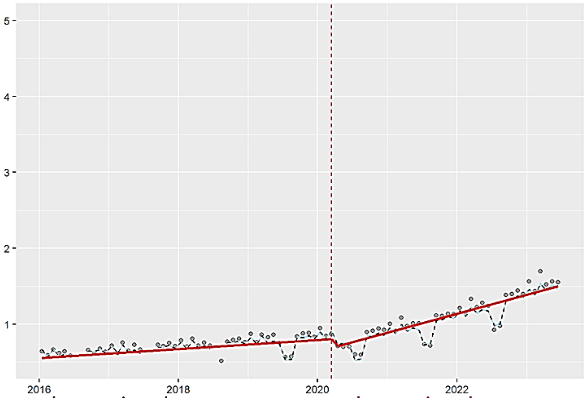

Increasing slope
RR **1.09 [1.08 – 1.10]****

Increasing slope
RR **1.26 [1.24 – 1.29]****

Differences between slopes
RR **1.15 [1.13 – 1.18]****

Men

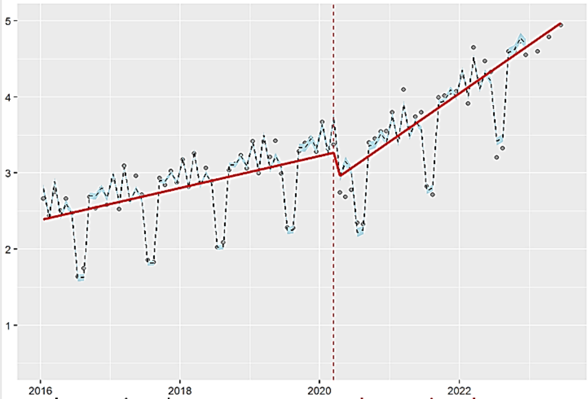

Increasing slope
RR **1.07 [1.06 – 1.08]****

Increasing slope
RR **1.17 [1.15 – 1.20]****

Differences between slopes
RR **1.09 [1.06 – 1.12]****

© 2024 Fond G et al. *JAMA Network Open*.

	Women	Men
Medications used in alcohol dependence	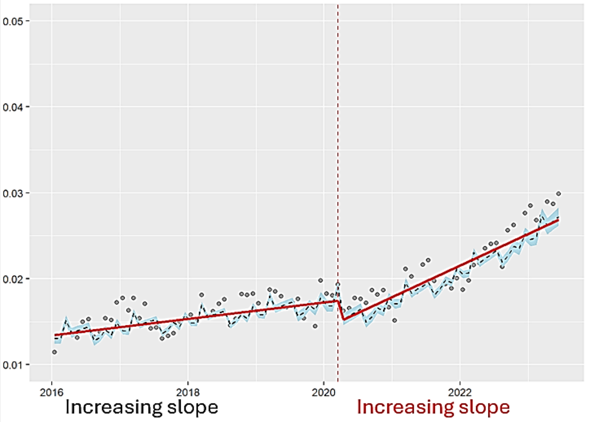<br>Increasing slope<br>RR **1.06 [1.03 – 1.09]****<br>Increasing slope<br>RR **1.19 [1.16 – 1.23]****<br>Differences between slopes<br>RR **1.12 [1.08 – 1.16]****	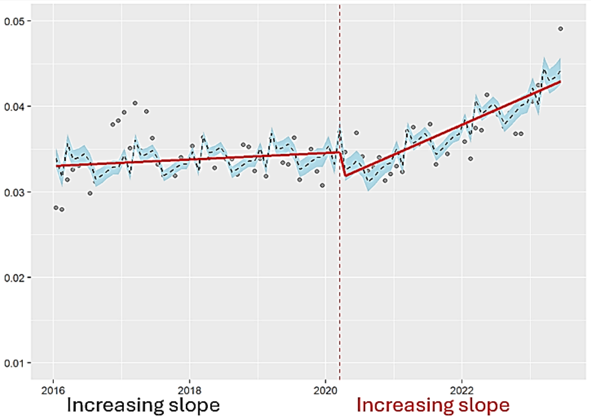<br>Increasing slope<br>RR **1.01 [0.98 – 1.04]**<br>Increasing slope<br>RR **1.09 [1.06 – 1.13]****<br>Differences between slopes<br>RR **1.08 [1.04 – 1.13]****

a: $p < 0.05$
b: $p < 0.001$
RR: relative risk.
Dotted lines are Quasi-Poisson estimates with confidence intervals. Dots are actual observations. The rates are expressed per 1,000 inhabitants.

© 2024 Fond G et al. *JAMA Network Open*.

eFigure 2. Age-Specific Trends in Mental Health Care and Prescriptions Before and After the Beginning of the COVID-19 Pandemic With Annual Relative Risk

	0-5 years	6-12 years	13-17 years	18-25 years
Outpatient psychiatric consultation	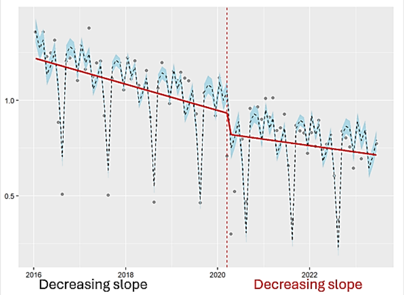<br>Decreasing slope RR **0.93 [0.91 – 0.96]****<br>Decreasing slope RR **0.95 [0.87 – 1.04]**<br>Differences between slopes RR **1.02 [0.91 – 1.13]**	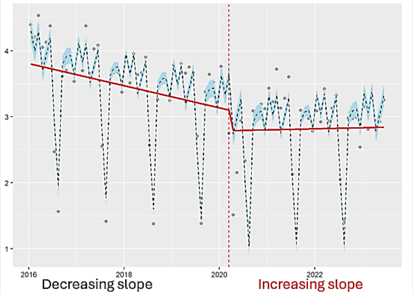<br>Decreasing slope RR **0.95 [0.93 – 0.97]****<br>Increasing slope RR **1.00 [0.93 – 1.08]**<br>Differences between slopes RR **1.05 [0.97 – 1.14]**	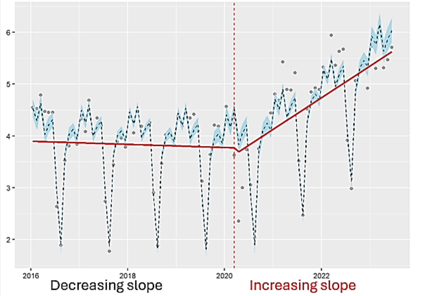<br>Decreasing slope RR **0.99 [0.97 – 1.01]**<br>Increasing slope RR **1.14 [1.06 – 1.21]****<br>Differences between slopes RR **1.15 [1.06 – 1.23]****	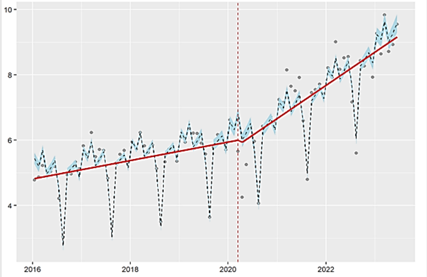<br>Increasing slope RR **1.05 [1.03 – 1.07]****<br>Increasing slope RR **1.14 [1.10 – 1.19]****<br>Differences between slopes RR **1.08 [1.03 – 1.14]****
Psychiatric full-time hospitalization	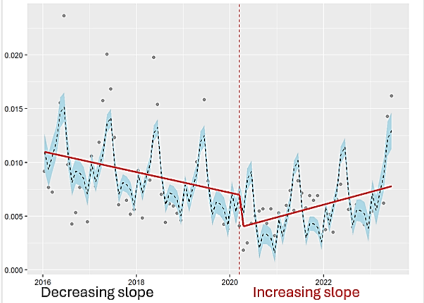<br>Decreasing slope RR **0.89 [0.84 – 0.95]****<br>Increasing slope RR **1.22 [0.98 – 1.52]**<br>Differences between slopes RR **1.36 [1.08 – 1.73]***	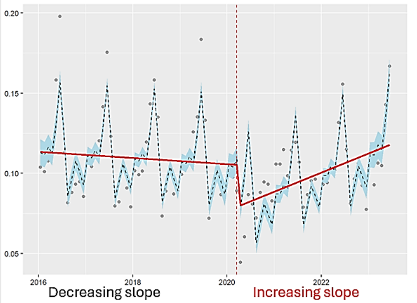<br>Decreasing slope RR **0.98 [0.95 – 1.01]**<br>Increasing slope RR **1.12 [1.00 – 1.27]***<br>Differences between slopes RR **1.14 [1.00 – 1.31]***	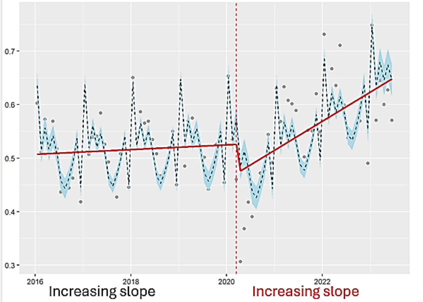<br>Increasing slope RR **1.00 [0.99 – 1.02]**<br>Increasing slope RR **1.10 [1.01 – 1.19]***<br>Differences between slopes RR **1.09 [1.00 – 1.19]***	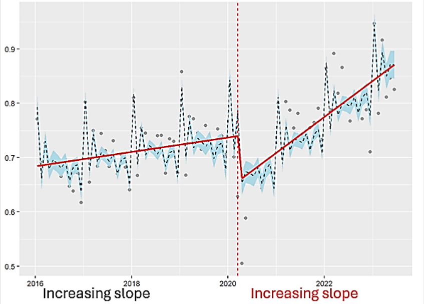<br>Increasing slope RR **1.01 [1.00 – 1.03]***<br>Increasing slope RR **1.09 [1.05 – 1.13]****<br>Differences between slopes RR **1.07 [1.02 – 1.12]***

© 2024 Fond G et al. *JAMA Network Open*.

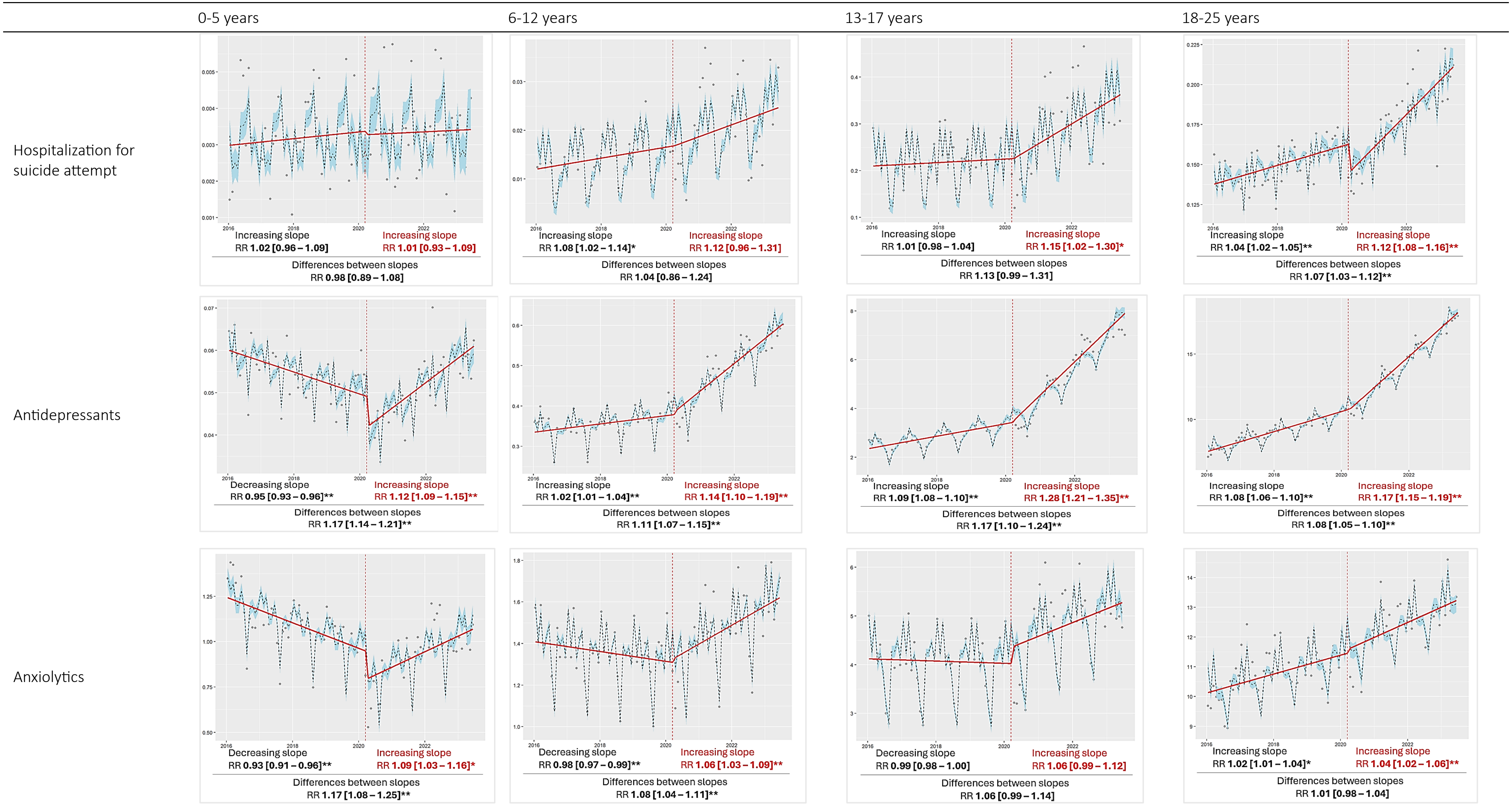

© 2024 Fond G et al. *JAMA Network Open*.

	0-5 years	6-12 years	13-17 years	18-25 years
Hypnotics	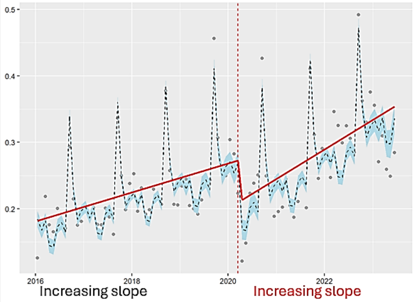<br>Increasing slope<br>RR 1.10 [1.06 – 1.13]**<br>Increasing slope<br>RR 1.17 [1.09 – 1.25]**<br>Differences between slopes<br>RR 1.06 [0.99 – 1.14]	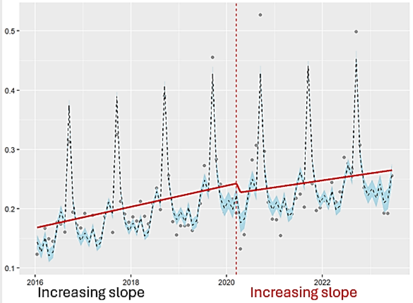<br>Increasing slope<br>RR 1.09 [1.06 – 1.12]**<br>Increasing slope<br>RR 1.04 [0.98 – 1.11]<br>Differences between slopes<br>RR 0.96 [0.89 – 1.03]	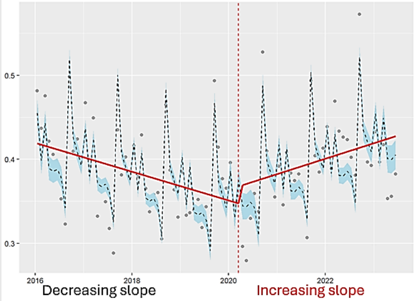<br>Decreasing slope<br>RR 0.95 [0.93 – 0.97]**<br>Increasing slope<br>RR 1.04 [1.00 – 1.09]*<br>Differences between slopes<br>RR 1.09 [1.04 – 1.15]**	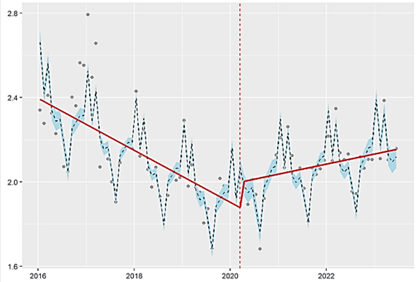<br>Decreasing slope<br>RR 0.94 [0.92 – 0.96]**<br>Increasing slope<br>RR 1.02 [1.01 – 1.03]**<br>Differences between slopes<br>RR 1.08 [1.06 – 1.10]**
Mood stabilizers	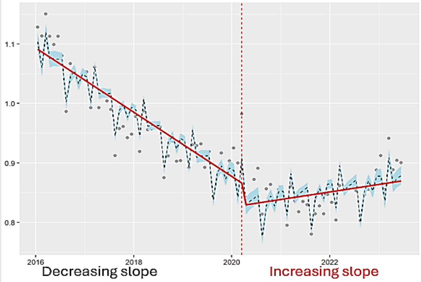<br>Decreasing slope<br>RR 0.94 [0.93 – 0.95]**<br>Increasing slope<br>RR 1.01 [0.99 – 1.03]<br>Differences between slopes<br>RR 1.07 [1.04 – 1.10]**	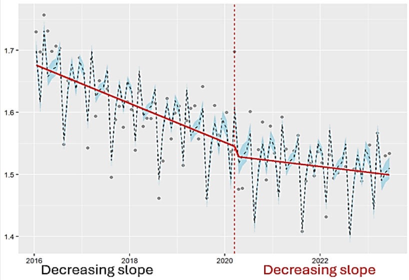<br>Decreasing slope<br>RR 0.98 [0.97 – 0.98]**<br>Decreasing slope<br>RR 0.99 [0.98 – 1.00]<br>Differences between slopes<br>RR 1.01 [1.00 – 1.02]*	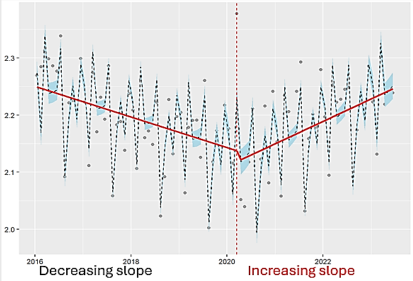<br>Decreasing slope<br>RR 0.98 [0.98 – 0.99]**<br>Increasing slope<br>RR 1.01 [1.01 – 1.02]**<br>Differences between slopes<br>RR 1.03 [1.02 – 1.03]**	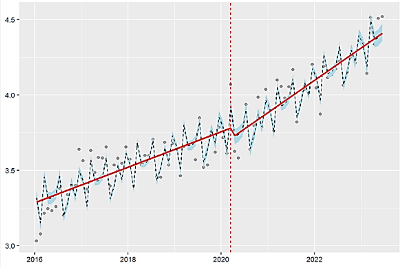<br>Increasing slope<br>RR 1.03 [1.02 – 1.04]**<br>Increasing slope<br>RR 1.05 [1.04 – 1.06]**<br>Differences between slopes<br>RR 1.01 [1.00 – 1.03]*
Antipsychotics	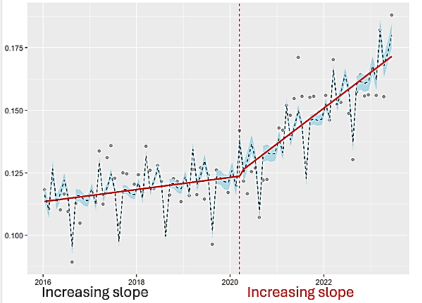<br>Increasing slope<br>RR 1.02 [1.00 – 1.03]*<br>Increasing slope<br>RR 1.10 [1.06 – 1.13]**<br>Differences between slopes<br>RR 1.07 [1.04 – 1.11]**	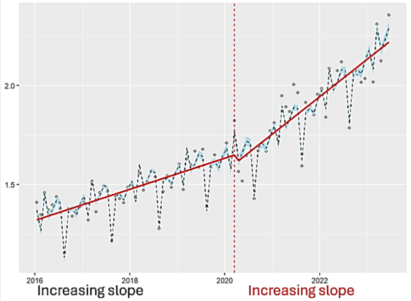<br>Increasing slope<br>RR 1.05 [1.04 – 1.06]**<br>Increasing slope<br>RR 1.10 [1.08 – 1.12]**<br>Differences between slopes<br>RR 1.04 [1.02 – 1.06]**	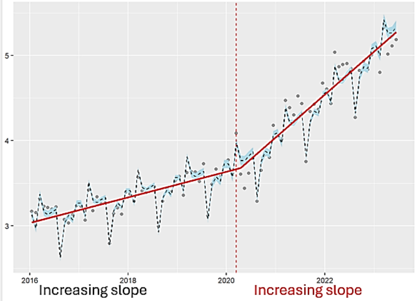<br>Increasing slope<br>RR 1.04 [1.03 – 1.05]**<br>Increasing slope<br>RR 1.12 [1.09 – 1.14]**<br>Differences between slopes<br>RR 1.07 [1.04 – 1.09]**	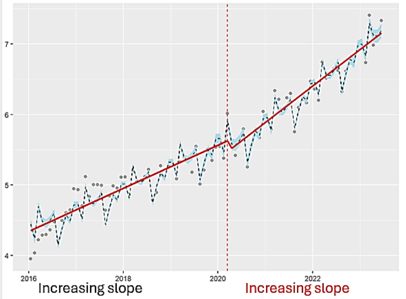<br>Increasing slope<br>RR 1.06 [1.04 – 1.08]**<br>Increasing slope<br>RR 1.08 [1.08 – 1.08]**<br>Differences between slopes<br>RR 1.02 [1.00 – 1.03]*

© 2024 Fond G et al. *JAMA Network Open*.

	0-5 years	6-12 years	13-17 years	18-25 years
Methylphenidate	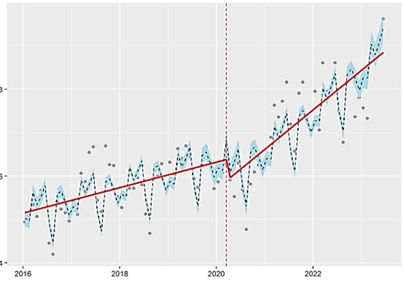<br>Increasing slope<br>RR **1.05 [1.03 – 1.07]****<br>Increasing slope<br>RR **1.13 [1.08 – 1.17]****<br>Differences between slopes<br>RR **1.07 [1.02 – 1.12]***	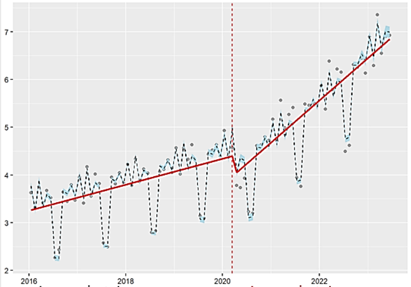<br>Increasing slope<br>RR **1.07 [1.06 – 1.08]****<br>Increasing slope<br>RR **1.18 [1.15 – 1.20]****<br>Differences between slopes<br>RR **1.09 [1.07 – 1.12]****	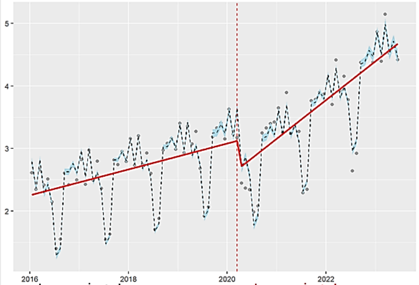<br>Increasing slope<br>RR **1.08 [1.06 – 1.09]****<br>Increasing slope<br>RR **1.18 [1.15 – 1.21]****<br>Differences between slopes<br>RR **1.09 [1.07 – 1.12]****	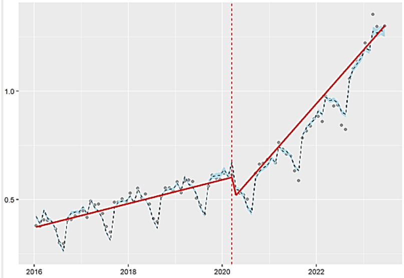<br>Increasing slope<br>RR **1.12 [1.10 – 1.13]****<br>Increasing slope<br>RR **1.33 [1.31 – 1.36]****<br>Differences between slopes<br>RR **1.19 [1.16 – 1.22]****
Medications used in alcohol dependence	NA	NA	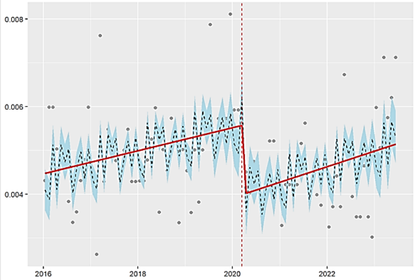<br>Increasing slope<br>RR **1.05 [0.99 – 1.12]**<br>Increasing slope<br>RR **1.08 [0.97 – 1.19]**<br>Differences between slopes<br>RR **1.02 [0.90 – 1.15]**	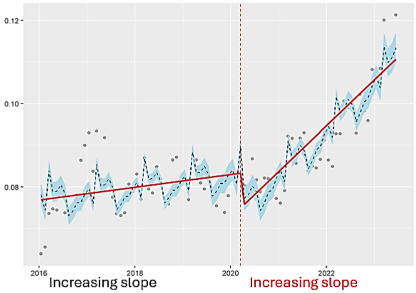<br>Increasing slope<br>RR **1.01 [0.98 – 1.05]**<br>Increasing slope<br>RR **1.12 [1.09 – 1.15]****<br>Differences between slopes<br>RR **1.10 [1.06 – 1.15]****

a: $p < 0.05$
b: $p < 0.001$
RR: relative risk.
Dotted lines are Quasi-Poisson estimates with confidence intervals. Dots are actual observations. The rates are expressed per 1,000 inhabitants.
NA: not applicable.

© 2024 Fond G et al. *JAMA Network Open*.
