# Supplementary material for: Mental Health Care Utilization and Prescription Rates Among Children, Adolescents, and Young Adults in France
Source: JAMA Netw Open. 2025 Jan 7;8(1):e2452789. doi: 10.1001/jamanetworkopen.2024.52789 (PMC11707635; doi:10.1001/jamanetworkopen.2024.52789)
Supplement: Supplement 2. — Data Sharing Statement [file jamanetwopen-e2452789-s002.pdf]

Data Sharing Statement

Fond. Mental Health Care Utilization and Prescription Rates Among Children, Adolescents, and Young Adults in France. *JAMA Netw Open*. Published December 30, 2024. doi:10.1001/jamanetworkopen.2024.52789

Data
Data available: No

Additional Information
Explanation for why data not available: national data from the French insurance database requiring an access code
